# Structural basis of synaptic vesicle assembly promoted by α-synuclein

**DOI:** 10.1038/ncomms12563

**Published:** 2016-09-19

**Authors:** Giuliana Fusco, Tillmann Pape, Amberley D. Stephens, Pierre Mahou, Ana Rita Costa, Clemens F. Kaminski, Gabriele S. Kaminski Schierle, Michele Vendruscolo, Gianluigi Veglia, Christopher M. Dobson, Alfonso De Simone

**Affiliations:** 1 Department of Chemistry, University of Cambridge, Cambridge CB2 1EW, UK; 2 Department of Life Sciences, Imperial College London, London SW7 2AZ, UK; 3 Department of Chemical Engineering and Biotechnology, University of Cambridge, Cambridge CB2 3RA, UK; 4 Department of Chemistry & Department of Biochemistry, Molecular Biology & Biophysics, University of Minnesota, Minneapolis, Minnesota 55455, USA; 5 Present address: Laboratory for Optics and Biosciences, Ecole Polytechnique, Palaiseau, 91128 Cedex, France

## Abstract

α-synuclein (αS) is an intrinsically disordered protein whose fibrillar aggregates are the major constituents of Lewy bodies in Parkinson's disease. Although the specific function of αS is still unclear, a general consensus is forming that it has a key role in regulating the process of neurotransmitter release, which is associated with the mediation of synaptic vesicle interactions and assembly. Here we report the analysis of wild-type αS and two mutational variants linked to familial Parkinson's disease to describe the structural basis of a molecular mechanism enabling αS to induce the clustering of synaptic vesicles. We provide support for this ‘double-anchor' mechanism by rationally designing and experimentally testing a further mutational variant of αS engineered to promote stronger interactions between synaptic vesicles. Our results characterize the nature of the active conformations of αS that mediate the clustering of synaptic vesicles, and indicate their relevance in both functional and pathological contexts.

## Abstract (web summary)

α-synuclein, a protein associated to Parkinson's disease, is involved in synaptic vesicle interaction and assembly. Here, the authors use NMR spectroscopy and super-resolution microscopy to unveil the nature and molecular mechanism of α-synuclein-mediated synaptic vesicle clustering.

## Graphical abstract

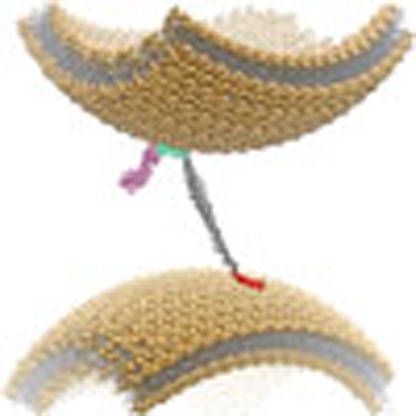

α-Synuclein (αS) is a 140-residue protein whose aggregation has been strongly associated with Parkinson's disease (PD)^1^,^2^,^3^,^4^,^5^. Fibrillar deposits of αS are the major constituents of Lewy bodies^6^,^7^,^8^, a hallmark of the disease, and inherited forms of early onset PD are associated with mutations, duplications and triplications of the αS-encoding gene^9^,^10^. Despite the general consensus on its pathological relevance, the physiological role of αS remains widely debated. In this context, a view is emerging in which αS is involved in the dynamics of synaptic vesicle (SV) trafficking by regulating a distal reserve pool of SVs that controls the amount of vesicles docked at the synapses during neurotransmitter release^11^,^12^. This biological role is directly associated with the ability of αS to bind to synaptic vesicles and induce their interaction and assembly *in vitro* and *in vivo*^13^,^14^,^15^,^16^. Indeed, in dopaminergic neurons αS exists in a tightly regulated equilibrium^17^ between a cytosolic monomeric form, which is predominantly disordered^18^,^19^,^20^, and a membrane-bound state, which is rich in α-helix structure in the region spanning residue 1–90 of the protein sequence^15^,^21^,^22^,^23^,^24^,^25^,^26^.

Because of its intrinsic ability to bind to a wide variety of biological membranes, the physiological state of membrane-bound αS is extremely difficult to characterize, as a variety of factors, including the presence of detergents^22^ and chemical modification of the protein^27^, can alter dramatically the structural properties of its bound state^15^. In a recent study, three major regions were identified to have distinct structural and dynamical properties that influence in different ways the nature of the membrane-bound state of αS^28^; these regions include an N-terminal α-helical segment, acting as the membrane-anchor, an unstructured C-terminal region, weakly associated with the membrane, and a central region, undergoing order–disorder transitions in the membrane-bound state and determining the affinity of αS for lipid bilayers of different composition^28^. This structural variability indicates that it is of fundamental importance to investigate the binding of αS to lipid membranes under conditions that reproduce as closely as possible the physiological environment relevant to that of presynaptic vesicles^15^.

We describe here a detailed characterization of the dynamical and structural properties at the surface of synaptic-like lipid vesicles of two familial αS mutations that have opposite effects on its affinity for membrane binding^29^,^30^. On the basis of these studies, we characterized the details of the underlying mechanism by which a single molecule of αS binds two different synaptic vesicles and promotes their interaction and assembly. This mechanism, which involves a double-anchoring step enabling αS to form a dynamic link between two vesicles, is strongly supported by an experiment in which a variant of αS was engineered to adopt structural properties in its membrane-bound state that result in enhanced αS-mediated interactions between synaptic vesicles while maintaining the same amino acid composition, charge and membrane-binding affinity of the wild-type protein. The mechanism, which was verified using both synthetic lipid vesicles and synaptic vesicles purified from rat brain, provides evidence that the specific level of affinity for membrane binding of the non-amyloid-β component (NAC) region of αS is a fundamental functional property enabling this protein to mediate the interaction between vesicles.

## Results

### Binding of αS variants to membranes

Using solution-state and solid-state nuclear magnetic resonance (NMR) spectroscopy in combination with cryo-electron microscopy (cryo-EM) and stimulated emission depletion (STED) imaging, we have characterized the structural properties at the surface of synaptic-like vesicles of the familial αS mutants A30P^31^ (αS_A30P_) and E46K^32^ (αS_E46K_) and compared their behaviour with that of the wild-type protein (αS_WT_)^28^. In particular, we studied the interactions of αS_A30P_, αS_E46K_ and αS_WT_ with small unilamellar vesicles (SUVs) composed of a mixture of 1,2-dioleoyl-sn-glycero-3-phosphoethanolamine (DOPE), 1,2-dioleoyl-sn-glycero-3-phospho-L-serine (DOPS), and 1,2-dioleoyl-sn-glycero-3-phosphocholine (DOPC) in 5:3:2 molar ratios^29^, as such SUVs have been reported to be good mimics of synaptic vesicles for composition and curvature^15^. The combination of magic angle spinning (MAS^33^,^34^) measurements in solid-state NMR (ssNMR^35^) and chemical exchange saturation transfer (CEST^28^,^36^,^37^,^38^,^39^,^40^, Supplementary Fig. 1) experiments in solution-state NMR used in this study has already been shown to be highly effective in probing interactions between αS and SUVs, enabling the degree of order and disorder in the membrane-bound state of αS_WT_ to be characterized^28^.

**Figure 1:**
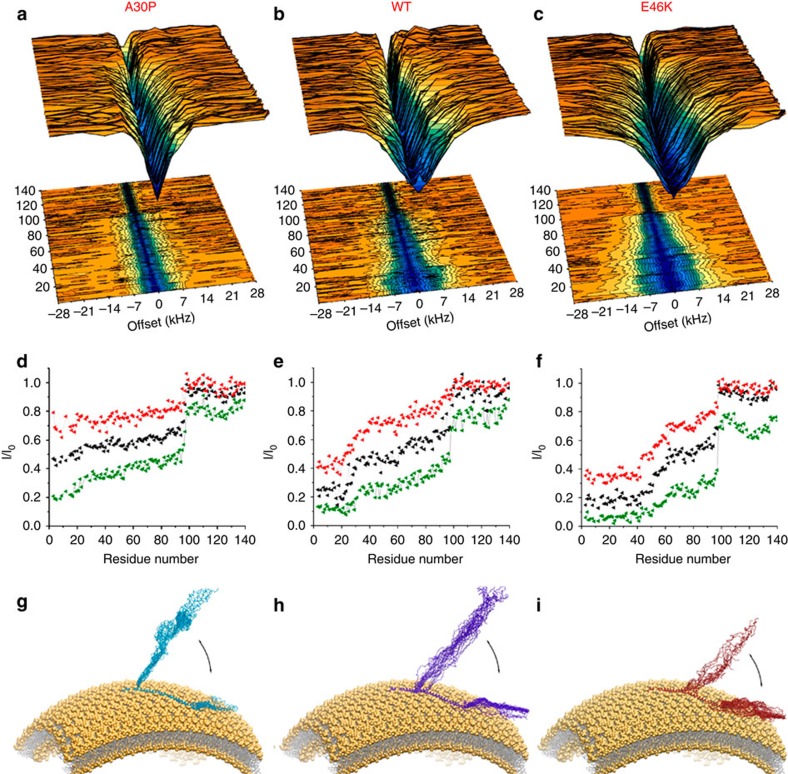
CEST experiments probing the membrane interactions of αS_A30P_ and αS_E46K_. CEST experiments were recorded at a ^1^H frequency of 700 MHz (see Methods section), using protein concentrations of 300 μM and 0.06% (0.6 mg ml^−1^) of DOPE:DOPS:DOPC lipids in a ratio of 5:3:2 and assembled into SUVs. ^1^H–^15^N HSQC spectra were recorded by using continuous wave saturation (170 Hz or 350 Hz) in the ^15^N channel at offsets ranging between −28 kHz and +28 kHz; an additional spectrum, saturated at −100 kHz, was recorded as a reference. Data recorded using a saturation bandwidth of 350 Hz are shown here (the data measured using a saturation bandwidth of 170 Hz are shown in Supplementary Fig. 2). For comparison, the plots in panels **b** and **e** are drawn using αS_WT_ data from our previous investigation^28^. (**a**–**c**) CEST surfaces for αS_A30P_ (**a**) αS_WT_^28^ (**b**) and αS_E46K_ (**c**). (**d**–**f**) CEST saturation along the sequences of αS_A30P_ (**d**), αS_WT_^28^ (**e**) and αS_E46K_ (**f**). The green lines refer to the averaged CEST profiles measured using offsets at +/− 1.5 kHz, and the profiles for +/− 3 kHz and +/− 5 kHz are shown in black and red, respectively. (**g**–**i**) Schematic illustration (see Materials and Methods) of the equilibrium between surface attached/detached local conformations in the membrane-bound states αS_A30P_ (**g**) αS_WT_^28^ (**h**) and αS_E46K_ (**i**). The major differences in the data of αS_A30P_, αS_WT_ and αS_E46K_ are located in the anchor region. Overall, these three variants of αS maintain the same topological properties at the surfaces of synaptic-like SUVs.

In the analysis of the interaction between αS_A30P_ and SUVs, CEST profiles (Fig. 1 and Supplementary Fig. 2) provided detailed information concerning the effects of the A30P mutation, which was shown to reduce very substantially the binding affinity of αS for SUVs^15^. In αS_A30P_, indeed, the binding of the N-terminal anchor region was found to involve a smaller number of residues than in the case of αS_WT_ (the N-terminal 20 residues compared with the N-terminal 25 residues in αS_WT_) with generally a lower degree of CEST saturation than in the case of αS_WT_. The major differences between αS_A30P_ and αS_WT_ were evident in this membrane-anchor N-terminal region, while the remainder of the protein sequence showed very limited variations in the CEST profiles of these two proteins. The opposite behaviour was observed for the αS_E46K_ variant, which binds SUVs with higher affinity than does αS_WT_^15^. Indeed, the CEST data indicate a significantly stronger interaction with the membrane for the N-terminal anchor region of αS_E46K_, which in this case extends up to residue 42 with a generally higher degree of saturation than in the case of αS_WT_. As with αS_A30P_, only marginal variations in the CEST profiles were observed in other regions of the sequence of αS_E46K_. The differences in the CEST saturation profiles of the two mutants compared with αS_WT_ (Supplementary Figs 3 and 4) indicate more specifically that the major changes in the modes of binding to SUVs of these mutational variants are associated primarily with the N-terminal region of the protein.

### Topology of αS_A30P_ and αS_E46K_ bound to SUVs

**Figure 2:**
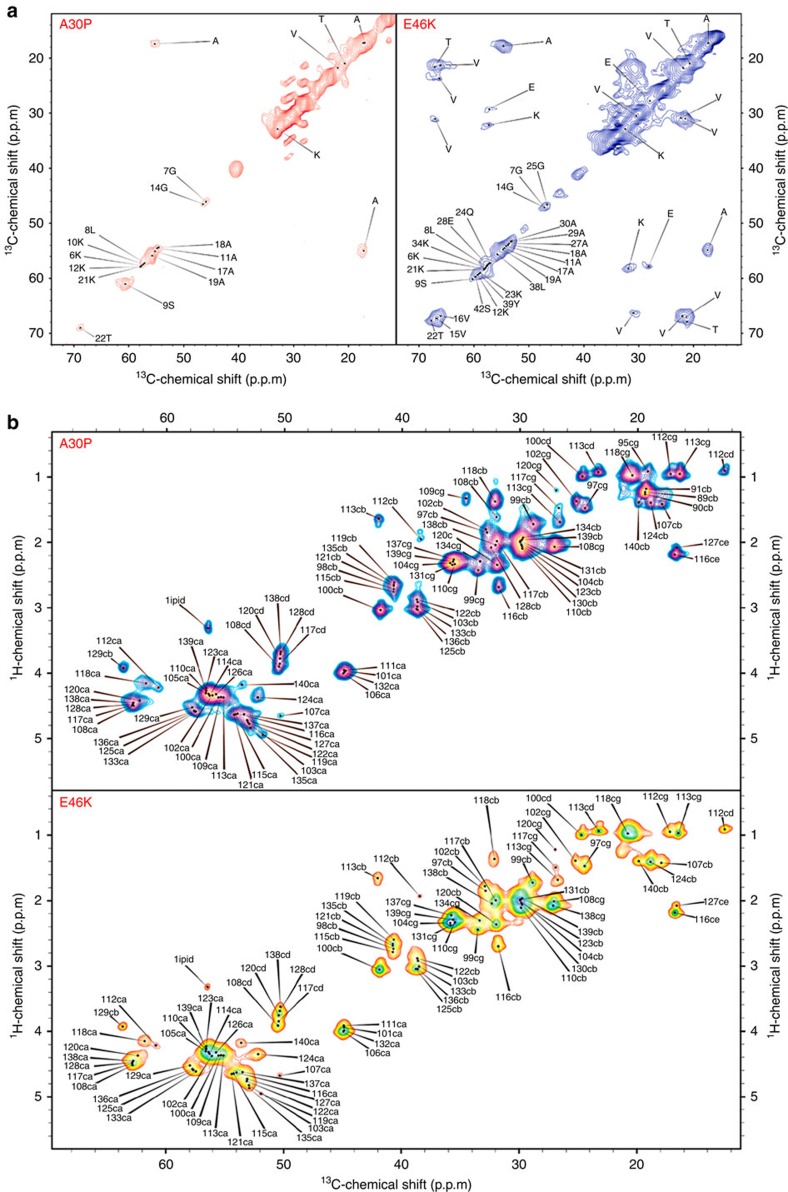
MAS ssNMR spectra of αS_A30P_ and αS_E46K_ bound to SUVs. (**a**) ^13^C–^13^C DARR correlation spectra (aliphatic regions) recorded at −19 °C using a 50 ms mixing time at a MAS rate of 10 kHz. We used a 1:65 protein:lipid ratio in both cases, and spectra of αS_A30P_ and αS_E46K_ are shown in the left and right panels, respectively. Residues are indicated using the single letter convention. The highest signal intensities in the spectra of the samples studied here were obtained by performing the measurements at −19 °C. Under these conditions the lipid mixtures used here are in the gel phase^70^, enabling ^13^C–^13^C DARR spectra to be measured with significantly increased signal-to-noise ratios but without affecting the pattern of chemical shifts; the latter are consistent with those measured at 4 °C (ref. 28). No variations in the number of observed resonances or in the chemical shifts were observed using protein:lipid ratios ranging from 1:30 to 1:200 (ref. 28). (**b**) ^1^H–^13^C correlation via INEPT transfer recorded at 4 °C at a MAS rate of 10 kHz. The experiments were performed at a ^1^H frequency of 700 MHz using a 3.2 mm E^Free^ probe. Atom names ca, cb, cg, cd and ce are used for C^α^, C^β^, C^γ^, C^δ^ and C^ɛ^ atoms, respectively.

To obtain detailed information on the topology of αS_A30P_ and αS_E46K_ when bound to the surface of SUVs, we used MAS ssNMR experiments. ^13^C–^15^N-labelled αS samples were mixed with SUVs, as described previously,^15^,^28^ to reach a protein:lipid ratio of 1:65 (ref. 28). Under these conditions we could observe directly the resonances of both rigid and dynamical regions of the membrane-bound αS molecule by using cross polarization and insensitive nuclei enhanced by polarization transfer (INEPT) experiments^41^, respectively. In the cross polarization regime, we performed ^13^C–^13^C dipolar-assisted rotational resonance (DARR)^42^ measurements to detect homonuclear correlations between carbon atoms of residues strongly anchored to the membrane (Fig. 2a). In our previous study of αS_WT_^28^, the ^13^C–^13^C DARR spectra identified resonances of residues 6–25 of the anchor region, showing that this region folds into a highly rigid α-helix lying essentially parallel to the membrane surface^28^. The ^13^C–^13^C DARR spectra of the membrane-bound states of αS_A30P_ and αS_E46K_ are, however, substantially different from those of αS_WT_, indicating that the dynamical and structural properties of the anchor region vary considerably between the wild-type and variant forms of αS.

In the case of αS_A30P_, the ^13^C–^13^C DARR spectrum showed a very limited signal-to-noise ratio and almost a complete absence of cross peaks, suggesting that the anchor region of this variant is significantly more dynamic than the same region of the wild-type protein. In contrast, the ^13^C–^13^C DARR spectrum of αS_E46K_ showed a higher signal-to-noise ratio and a significantly larger number of intense cross peaks, indicating an elongated anchor region in this mutational variant that binds more strongly to SUVs. Using the dipolar connectivities from ^15^N–^13^C cross polarization-based experiments, along with our previous assignment of αS_WT_ and ^13^C–^13^C DARR spectra acquired at different mixing times, we were able to assign individual spin systems in the ^13^C–^13^C DARR spectra of αS_A30P_ and αS_E46K_ (Fig. 2a). The chemical shifts were then compared with those obtained from solution-state NMR studies of αS_WT_ in SDS and SLAS micelles^22^,^43^, and indicate that, despite the differences in dynamics and in the binding strength relative to lipid membranes, all the variants analysed here adopt a helical conformation at the N-terminal anchor when bound to membranes. This finding is particularly relevant in the case of the A30P mutation as it shows that, like the other variants and despite the insertion of a helix-breaker residue, αS_A30P_ binds the lipid bilayer by adopting an amphipathic α-helix conformation at the N terminus and not as a disordered state that is tethered onto the lipid surface.

The highly dynamical regions of αS_A30P_ and αS_E46K_ bound to SUVs were then probed directly by INEPT measurements acquired using MAS ssNMR experiments^41^ and revealed ^1^H–^13^C correlations for resonances of the disordered C-terminal region of αS (Fig. 2b), which is only weakly associated with the membrane^28^. In contrast to the results obtained from the DARR spectra, no significant differences were found between αS_A30P_ and αS_E46K_ in the INEPT spectra. These measurements also indicated that the disordered C-terminal regions (residues 98–140) of the membrane-bound states of both variants have similar structural and dynamical properties to those of αS_WT_. To gain further insights into the topological nature of mobile regions in the membrane-bound αS_A30P_ and αS_E46K_ variants, we used paramagnetic relaxation enhancement experiments^28^. By doping the SUVs with low levels (2%) of a lipid carrying an unpaired electron on the head group, namely the gadolinium salt of PE-DTPA (1,2-dimyristoyl-sn-glycero-3-phosphoethanolamine-N-diethylenetriaminepentaacetic acid), we could observe selective line broadening of individual resonances in the INEPT spectrum of both variants (Supplementary Fig. 5), revealing those residues that interact transiently with the membrane surface. The resulting broadening patterns of hydrophobic (including L100, A107, P108, I112, L113, P117, V118 and P120) and positively charged (K97 and K102) residues were found to be similar to those observed in αS_WT_. The selective broadening of the INEPT peaks of the C-terminal residues in these paramagnetic relaxation enhancement experiments is in agreement with the modest levels of saturation transfer that are detected for this region using CEST experiments performed with a bandwidth of 350 Hz and offset frequencies of ±1.5 kHz (Supplementary Fig. 4a), which also provide evidence for transient tethering of the C-terminal region of αS onto the membrane surface.

### Mechanism of vesicle assembly promoted by αS

**Figure 3:**
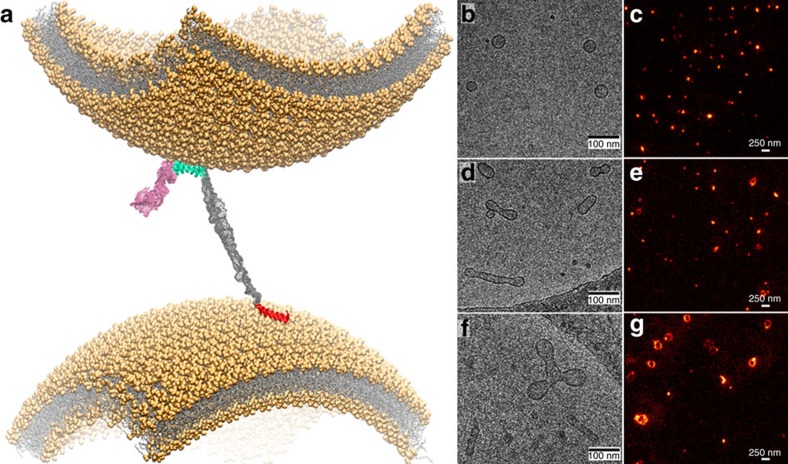
Vesicle assembly induced by αS. (**a**) Molecular details of the double-anchor mechanism described in this work. SUVs of 50 nm in diameter were modelled to mimic as closely as possible the experimental conditions in this study (see Methods section). αS was modelled with the N-terminal anchor in an amphipathic α-helical conformation (red) and bound to the lower vesicle. The region 65–97 (cyan) of αS was modelled in an amphipathic α-helical conformation bound to the upper vesicle. The C-terminal fragment (residues 98–140) and the linker region 26–59 are shown in pink and grey colours, respectively. With this topology the modelling reveals that a single αS molecule could simultaneously bind two vesicles that are up to 150 Å apart. (**b**,**c**) Cryo-EM (**b**) and STED (**c**) images acquired on SUVs at a concentration of 0.5 mg ml^−1^. (**d**,**e**) Cryo-EM (**d**) and STED (**e**) images measured on SUVs following a 12 h incubation with 200 μM αS_WT_. (**f**,**g**) Cryo-EM (**f**) and STED (**g**) images acquired on SUVs following a 12 h incubation with 200 μM αS_Sw_.

The solution-state and solid-state NMR measurements described above reveal a striking degree of independence between the membrane-binding properties of the N-terminal membrane-anchor region of αS, which is significantly affected by the A30P and E46K mutations, and of the region spanning residues 65–97, which instead shows negligible differences as a result of these mutations (Supplementary Figs 3 and 4). These independent membrane-binding modes suggest that, in addition to interacting with the membrane surface of the same SUV, these two regions are sufficiently independent to bind simultaneously two different SUVs. Indeed, our modelling studies show that a single αS molecule could bind and bridge two vesicles that are as much as 150 Å apart (Fig. 3a), with both the N-terminal anchor region (the N-terminal 25 residues) and the central region of αS (residues 65–97) adopting the conformations of amphipathic α-helices. These data, therefore, provide the structural basis of the mechanism by which αS promotes the interaction between vesicles that has been observed experimentally both *in vitro*^14^,^15^ and *in vivo*^13^,^16^.

To obtain further evidence of this ‘double-anchor' mechanism (Fig. 3a), we used our findings to design a further variant of αS having structural properties that we anticipated should enhance the probability of αS binding simultaneously to two different vesicles in such a way as to mediate their interaction. In particular, to favour the double-anchor mechanism (Fig. 3a), this variant was designed to enhance the detachment of the region 65–97 from the membrane surface when αS is bound to the SUVs via its N-terminal anchor region. We identified for this purpose a swapped sequence (αS_Sw_) incorporating the E46K and K80E mutations (Supplementary Fig. 6). In particular, by replacing the lysine at position 80 with a glutamic acid (K80E), the local binding to negatively charged vesicles is disfavoured, hence shifting the conformational equilibrium of the fragment 65–97 towards a state where this region is less strongly bound to the SUV surface. By contrast, because the K80E mutation also affects the overall membrane affinity of αS, a second mutation in which the glutamic acid at position 46 is replaced by a lysine (E46K) was selected to increase the interaction between the N-terminal anchor region and the SUV surface, as probed in αS_E46K_ (Figs 1 and 2), thereby restoring an overall *K*_*D*_ comparable to that of the wild-type protein.

We tested experimentally whether or not αS_Sw_ possessed the anticipated structural and thermodynamical properties characteristic of its membrane-bound state. In agreement with our design, we found the binding affinity of αS_Sw_ for SUVs, measured by circular dichroism^28^, to be similar to that of αS_WT_ (Supplementary Fig. 7a–c). By contrast the structural properties of the αS_Sw_ variant, as probed by CEST (Supplementary Fig. 7d–f), showed a significant reduction in the membrane interaction of the central region (residues 65–97) of the variant than in αS_WT_. These data indicate that αS_Sw_ binds SUVs with essentially the same overall affinity as αS_WT_ but assumes different structural and dynamical properties in its bound state that promote an enhanced exposure of the segment 65–97. CEST also confirmed the stronger interaction of the anchor region of αS_Sw_ compared with that of αS_WT_, which in the designed variant is extended to residue 42 as a consequence of the E46K mutation (Supplementary Fig. 7e). As αS_Sw_ and αS_E46K_ have the same sequence except at position 80, we plotted the differences in the CEST profiles of these two variants; this comparison reveals clearly that the binding properties of these two variants to the SUVs are indistinguishable except in the region 65–97 (Supplementary Fig. 8) thereby providing additional evidence for the independence of the membrane-binding properties of the N-terminal and central regions in αS.

### Synaptic vesicle assembly induced by αS_WT_ and αS_Sw_

We compared the efficiency with which αS_Sw_ and αS_WT_ promote the interaction and assembly of vesicles by monitoring, using cryo-EM, the ability of the two variants to promote coalescence and fusion of synaptic-like SUVs *in vitro*^16^. As a control, cryo-EM images of 0.05% DOPE:DOPS:DOPC SUVs incubated for 12 h in the absence of αS showed spherical vesicles of diameters ranging between 30 and 60 nm, with negligible evidence of vesicle fusion over the period of incubation (Fig. 3b and Supplementary Fig. 9a), showing that in the absence of αS the spontaneous fusion of SUVs occurs with extremely slow kinetics. In the presence of αS, however, the SUVs experience a considerable number of fusion events, with cryo-EM images revealing the presence of elongated fused vesicles having long axes of up to 200 nm under the conditions used in this study (Fig. 3d and Supplementary Fig. 9b). Incubating SUVs with αS_Sw_, however, resulted in a very significant increase in the extent of vesicle fusion, with cryo-EM images clearly indicating that this variant is significantly more active in promoting the interaction of SUVs ultimately leading to very large vesicle assemblies (Fig. 3f and Supplementary Fig. 9c).

To generate a quantitative analysis of the sizes of the SUVs in the presence and absence of the αS variants, we used STED microscopy, which enabled us to sample rapidly the sizes of thousands of vesicles to achieve statistically significant differences in the distributions. STED imaging was carried out by fluorescently labelling the DOPE:DOPS:DOPC SUVs with 2% of DOPE labelled with the ATTO 647N fluorophore. Images of isolated vesicles resulted in a size distribution centred at 55±11 nm (Fig. 3c and Supplementary Fig. 10a–b), within the range anticipated from the preparation protocol^28^. However, STED images of vesicles incubated with αS_WT_ clearly revealed the presence of numerous clusters of fused lipids, whose sizes were quantified by using an algorithm that fits annular shapes to the lipid vesicles, rather than the 2D Gaussian fitting used in the case of isolated vesicles (Methods section and Supplementary Fig. 10). The resulting distributions indicate that SUVs incubated with αS_WT_ give rise to two populations of vesicles, one with properties similar to those of vesicles imaged in the absence of αS and another attributable to fused vesicles, with a distribution centred at 115±30 nm and with a relative population of 16% of the imaged vesicles (Fig. 3e and Supplementary Fig. 10c–d).

**Figure 4:**
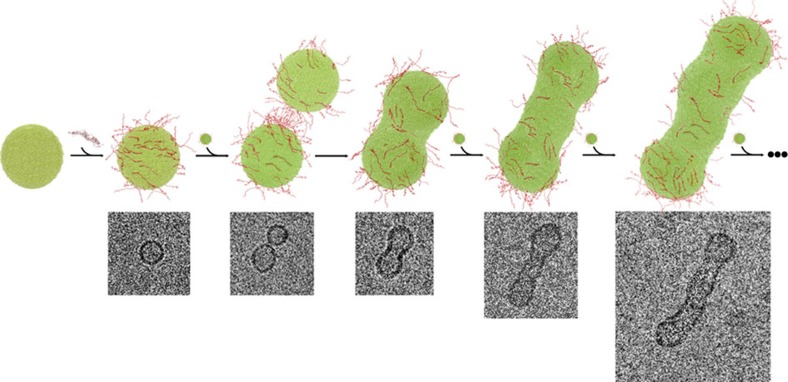
Stepwise representation of SUV interactions and fusion promoted by αS. The scheme shows the stepwise mechanism of vesicles assembly as probed from images obtained *in vitro* by cryo-EM, which are also shown. Disordered cytoplasmatic αS (red) binds dynamically to the surface of SUVs (green), as described in this study. SUVs coated with αS assemble with fast kinetics as a consequence of the double-anchor mechanism promoted by the αS molecules decorating their surfaces. The tethered SUVs, which are initially assembled together in dimeric, trimeric, tetrameric and higher order states, eventually fuse to form larger vesicles. With the increasing size of the fused vesicle, we observed preferential fusion events at the termini of the aggregated vesicles. This observation can be explained by the higher affinity of αS for significantly curved membrane surfaces^19^, which increases the concentration of bound αS at the termini of the elongated vesicles thereby promoting a stronger double-anchor mechanism in these loci.

Following incubation with αS_Sw_, however, both the sizes and relative populations of the fused vesicles increased markedly, with the size distribution of fused vesicles now centred at 181±48 nm and with a relative population of 32% of the imaged vesicles (Fig. 3g and Supplementary Fig. 10e–f). Similar conclusions to those obtained from STED analysis were obtained from measurements of dynamic light scattering (DLS), which showed that the average size of the SUVs increases in the presence of αS and that this effect is considerably greater with αS_Sw_ than with αS_WT_ (Supplementary Fig. 11). Overall, the cryo-EM, STED and DLS data show that the engineered αS_Sw_ variant has a very significantly enhanced activity in promoting the interactions between SUVs. As αS_Sw_ and αS_WT_ have the same amino acid composition and net charge, and bind SUVs with similar thermodynamic affinity, the enhanced interactions between vesicles on incubation with αS_Sw_ can be attributed to the higher population of conformations with an enhanced exposure of the region 65–97 from the membrane surface, which increases the probability of associating with a different vesicle and to mediate the vesicle assembly via a double-anchor mechanism (Fig. 3a), as probed by the fusion of DOPE:DOPS:DOPC SUVs on incubation with the protein (Fig. 4).

**Figure 5:**
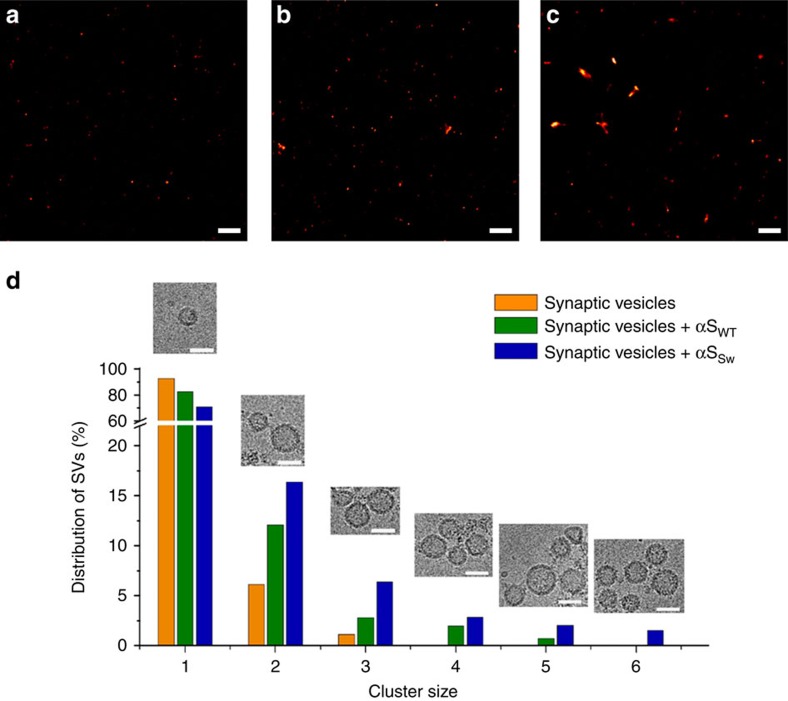
Clustering of synaptic vesicles promoted by αS. SVs purified from rat brain were incubated for 48 h at 37 °C. The concentrations during the incubation were 0.5 mg ml^−1^ and 85 μM for the SVs and the αS variants, respectively. (**a**–**c**) dSTORM imaging of SVs alone (**a**) and SVs incubated with αS_WT_ (**b**) and with αS_Sw_ (**c**). The images were collected using a previously described protocol^46^. Scale bars, 1 μm. To generate fluorescent SVs, we used a primary antibody that is specific for synaptotagmin 1 and a secondary antibody that is covalently linked to an ATTO 647 N dye. 10,000 fluorescence frames with an exposure time of 10 ms were recorded. The field of view imaged covered 1,997 × 1,997 camera pixels, corresponding to an area on the sample of ∼20 × 20 μm^2^. (**d**) To assess the level of clustering of the SVs, we adapted an approach that has previously been successfully employed to analyse protein self-assembly.^47^ For each dSTORM image, clusters of SVs were identified on the basis of the distances between the centres of mass of the SVs. In particular two or more vesicles were associated with a specific cluster if their distances apart are less than 60 nm. The distribution of SVs in clusters of different sizes is reported using orange, green and blue histograms for SVs, SVs in the presence of αS_WT_ and SVs in the presence of αS_Sw_, respectively. Cryo-EM images (scale bar, 50 nm) show representative clusters of different size.

To assess the role of the double-anchor mechanism in the clustering of synaptic vesicles (SVs) induced by αS, we incubated SVs purified from rat brain^44^,^45^ for 48 h at 37 °C in the presence or absence of αS samples (αS_WT_ or αS_Sw_). The distribution of the sizes of the clusters of SVs on incubation were quantified using direct stochastic optical reconstruction microscopy (dSTORM)^46^ to acquire images on poly-L-lysine-coated glass plates. To visualize SVs, we used a primary antibody that is specific for the synaptic protein synaptotagmin 1 and therefore binds selectively to SVs, and a secondary antibody fluorescently labelled with ATTO 647N. The resulting dSTORM images (Fig. 5a–c) were analysed by identifying the centres of mass of each of the SVs and then by applying a clustering approach^47^ to identify groups of SVs that, according to a distance cutoff of 60 nm, belong to the same cluster. The resulting distribution of sizes of SV clusters (Fig. 5d) showed that 93% of the vesicles remain isolated after incubation for 48 h at 37 °C in the absence of αS, with the remaining 6% and 1% assembled in clusters consisting of two and three SVs, respectively. After incubation under the same conditions but in the presence of 85 μM of αS_WT_, up to 17% of the vesicles were clustered, some including assemblies composed up to five SVs (1%). In the presence of αS_Sw_, however, over 29% of the vesicles were observed to be clustered up to six SVs (2%). Cryo-EM images of the assembled structures (Fig. 5d) revealed that the surfaces of the SVs composing the clusters are separated by distances that extend up to 15 nm, in agreement with the double-anchor mechanism (Fig. 3a).

## Discussion

It is widely recognized that the physiological activity of αS is associated with its ability to bind to a variety of lipid membranes^48^. A number of studies support an emerging view that a key biological role of αS is to mediate the interactions and assembly of synaptic vesicles^14^,^16^. Vesicle clustering by αS has been shown to be a vital step in many functional processes, including endoplasmic reticulum-to-Golgi vesicle trafficking^13^,^49^ and recycling of the SVs within the mechanism of neuronal communication^11^. In the present study, we have examined the structural principles underlying the αS-induced interactions and assembly of SVs by characterizing the membrane-binding properties of two mutational variants of αS linked with familial PD. This analysis reveals that two key regions of the protein, namely the N-terminal membrane-anchor (residues 1 to 25) and the central segment of the sequence (residues 65–97, having significant overlap with the NAC region), have independent membrane-binding properties and therefore are not only able to interact with a single SV, but can also simultaneously bind to two different vesicles (Fig. 3a) thereby promoting their interaction and assembly as shown *in vitro* and *in vivo*^14^,^15^,^16^. The resulting double-anchor mechanism explains why the deletion of the segment 71–82 in the NAC region of αS or the impairment of the membrane affinity of the N-terminal anchor region of the protein severely affects *in vivo* vesicle clustering as shown in *Saccharomyces cerevisiae*^16^. This mechanism also provides a structural explanation for the suggested loss of function caused by A30P, which is associated with an impairment of vesicle clustering by αS_A30P_ as observed both *in vitro* and *in vivo*^14^,^16^, as well as for the functional regulation proposed to occur via the phosphorylation of serine 87 (ref. 50). In particular, by introducing a negative charge in the region 65–97, the phosphorylation of S87, which has been identified both in functional contexts and in the case of synucleinopathies, has similar effects to those of the K80E mutation in αS_Sw_.

We tested this molecular mechanism by engineering the mutational variant αS_Sw_, which was designed to enhance the probability of αS binding simultaneously to two different vesicles. Our studies of this variant have provided strong evidence in support of the proposed mechanism by showing that enhanced exposure of the central region, spanning residues 65–97 in the membrane-bound state of αS, promotes more strongly the clustering of SVs purified from rat brain (Fig. 5) and the assembly and fusion of DOPE:DOPS:DOPC SUVs (Fig. 4). It has previously been suggested that a broken α-helix structural topology of αS^22^,^51^, which is a conformation that αS adopts on binding to detergent micelles, could play a role in vesicle-vesicle interactions stimulated by αS^48^,^52^. The present study, however, shows experimentally that the underlying mechanism by which αS mediates the interactions between lipid vesicles relies on the balance between ordered (membrane-bound) and disordered (membrane-detached) conformational states of the region spanning residues 65–97 of the protein. Perturbing this balance, as we have done rationally with αS_Sw_, or on alteration of the expression levels of αS, can dramatically affect its ability to promote the vesicle assembly *in vivo* leading to defects in the regulation of vesicle trafficking^11^,^13^,^49^,^53^,^54^,^55^,^56^,^57^.

Other studies also suggest that αS could act as a molecular chaperone for the formation of SNARE complexes, which appears to result from the direct interaction between αS and synaptobrevin 2 at the surface of SVs^14^,^58^. Such an interaction was shown to be independent of the NAC region, suggesting that this region has no direct functional role in this particular process^59^. The present data, however, reveal that the NAC region is not only involved in αS aggregation, as extensive evidence has previously indicated^4^,^60^,^61^, but also has a specific role in a key molecular mechanism associated with the normal function of αS. This study provides evidence that the membrane affinity of the NAC region of αS is finely tuned to ensure an optimal degree of local detachment from the membrane surface to enable binding to occur between different vesicles. The present finding that, by perturbing this fine tuning through the design of the αS_Sw_ variant, it is possible to promote stronger interactions between vesicles (Figs 3 and 5) indicates that the exposure of the region 65–97 in the vesicle-bound state of αS is crucial for the physiological mechanism of SVs clustering and, at least in the case of αS_Sw_, has more relevance than the local membrane-binding affinity of this region, which in this variant is reduced as a result of the K80E mutation. The selection toward sequence properties of αS that enable the detachment of the amyloidogenic NAC region from the vesicle surface to favour the functional mechanism described in this study, however, can also lead to aberrant behaviour, as these conformational states are particularly vulnerable to self-association leading to αS aggregation at membrane surfaces^11^,^62^,^63^,^64^,^65^. Taken together, these findings provide therefore a new mechanistic link between functional and pathological roles of αS.

## Methods

### αS purification

αS_WT_ was expressed and purified in *Escerichia coli* using a pT7-7 plasmid in which αS gene is under the control of the phage T7 RNA polymerase promoter. BL21 (DE3)-gold competent cells (Agilent Technologies, Santa Clara, USA) were transformed with this plasmid using heat-shock and subsequently grown in an isotope-enriched M9 minimal medium containing 1 g l^−1^ of ^15^N ammonium chloride, 2 g l^−1^ of ^13^C-glucose and 100 μg ml^−1^ ampicillin (Sigma-Aldrich, St Louis, USA) to produce uniformly ^15^N and/or ^13^C labelled αS samples. Cell growth was carried out at 37 °C under constant shaking at 250 rpm to an OD600 of 0.6. Subsequently the expression of the protein was induced with 1 mM isopropyl β-D-1-thiogalactopyranoside at 37 °C for 4 h and cells were then harvested by centrifugation at 6,200*g* (Beckman Coulter, Brea, USA). The cell pellets were resuspended in lysis buffer (10 mM Tris-HCl pH 8, 1 mM EDTA and EDTA-free complete protease inhibitor cocktail tablets obtained from Roche, Basel, Switzerland) and lysed by sonication. The cell lysate was centrifuged at 22,000*g* for 30 min to remove cell debris and the supernatant was then heated for 20 min at 70 °C and subsequently centrifuged at 22,000*g* to precipitate the heat-sensitive proteins. Subsequently streptomycin sulfate was added to the supernatant to a final concentration of 10 mg ml^−1^ to stimulate DNA precipitation. The mixture was stirred for 15 min at 4 °C followed by centrifugation at 22,000*g*. Then, ammonium sulfate was added to the supernatant to a concentration of 360 mg ml^−1^ to precipitate the protein. The solution was stirred for 30 min at 4 °C and centrifuged again at 22,000*g*. The resulting pellet was resuspended in 25 mM Tris-HCl, pH 7.7 and dialyzed against the same buffer to remove salts. The dialyzed solutions were then loaded onto an anion exchange column (26/10 Q sepharose high performance, GE Healthcare, Little Chalfont, UK) and eluted with a 0 to 1 M NaCl step gradient. αS was eluted at ∼300 mM NaCl and then further purified by loading onto a size-exclusion column (Hiload 26/60 Superdex 75 preparation grade, GE Healthcare, Little Chalfont, UK). All the fractions containing the monomeric protein were pooled together and concentrated by using Vivaspin filter devices (Sartorius Stedim Biotech, Göttingen, Germany). The purity of the aliquots after each step was analysed by SDS–PAGE and the protein concentration was determined from the absorbance at 275 nm using an extinction coefficient of 5,600 M^−1^ cm^−1^.

To express and purify the mutational variants of αS (αS_A30P_, αS_E46K_ and αS_Sw_), we employed the same experimental procedure as used in the case of αS_WT_. Single point mutations of the αS_WT_ plasmid were obtained using the Q5 Site-Directed Mutagenesis Kit (New England Biolabs, Inc., Ipswich, USA). Table 1 reports the primers employed to obtain the plasmids of αS_A30P_, αS_E46K_ and αS_Sw_.

### Preparation of synaptic-like SUVs

SUVs containing a molar ratio of 5:3:2 of DOPE:DOPS:DOPC (Avanti Polar Lipids, Inc., Alabaster, USA) were prepared from chloroform solutions of the lipids as described previously^15^,^28^. Briefly, the lipid mixture was evaporated under a stream of nitrogen gas and then dried thoroughly under vacuum to yield a thin lipid film. The dried thin film was re-hydrated by adding aqueous buffer (20 mM sodium phosphate, pH 6.0) at a concentration of 15 mg ml^−1^ (1.5%) and subjected to vortex mixing. In all NMR experiments described in this paper SUVs were obtained by using several cycles of freeze-thawing and sonication until the mixture became clear^15^,^28^. In the particular case of CEST experiments, SUVs at a concentration of 0.06% (0.6 mg ml^−1^) were mixed with αS samples after sonication. For ssNMR studies αS was added to the SUV mixtures up to a molar ratio of 1:65 protein:lipid. The mixtures were then pelleted at 300,000*g* for 30 min at 4 °C (Beckman Coulter Optima TLX Inc. Brea, USA) by using a TLA 100.3 rotor. Subsequently the SUV-αS samples were transferred into 3.2 mm Zirconia XC thin-walled MAS rotors for ssNMR experiments. For STED and cryo-EM imaging experiments, as well as for DLS, DOPE:DOPS:DOPC SUVs were prepared by extrusion through membranes with a 50 nm pore diameter (Avanti Polar Lipids, Inc) after re-hydration in 20 mM sodium phosphate (pH 6.0) at a concentration of 1.0 mg ml^−1^ (0.1%).

### Purification of SVs from rat brain

SVs composed of phospholipid molecules (amounting to 30% of their composition), proteins (58%) and cholesterol (12%)^44^,^45^ were purified from brains of rat provided by Charles River Laboratories (Animal work was approved by the Named Animal Care and Welfare Officer (NACWO) and the Ethics Review Committee of the University of Cambridge). Rat brains were homogenized in 4 mM HEPES and 320 mM sucrose buffer using protease inhibitors *via* ten strokes at 900 r.p.m. in a glass-Teflon homogenizer (Wheaton, Millville, USA)^44^,^45^. All steps were carried out at 4 °C or in ice. The homogenates were centrifuged at 1,000*g* for 10 min and the supernatants were collected and further centrifuged at 15,000*g* for 15 min. The supernatants from the second centrifugation (Sup-2) were stored at 4 °C. The pellets from the second centrifugation, which contained the synaptosomes, were lysed using ice-cold water by applying three strokes at 2,000 r.p.m. Subsequently, HEPES buffer solutions containing protease inhibitors were added to the lysates and the resulting solutions were centrifuged at 17,000 *g* for 15 min, and the supernatant was combined with the Sup-2 supernatants. The resulting mixtures were centrifuged at 48,000*g* for 25 min and the supernatants were loaded onto a 0.7 M sucrose cushion and centrifuged at 133,000*g* for 1 h. The bottom half of the sucrose cushion was pooled and centrifuged at 300,000*g* for 2 h. The pellets were resuspended in buffer (100 mM Tris-HCl, pH 7.4, 100 mM KCl) and loaded onto a Sephacryl S-1000 size-exclusion chromatography column (100 × 1 cm) resulting in a distinctive peak of the SVs^44^,^45^. The SVs were then stained against specific SV antibodies, including synaptotagmin 1, synaptobrevin 2, by western blot^44^,^45^. To perform dSTORM analyses, the SVs were incubated with a primary antibody (dilution 1:1000) that specifically recognizes synaptotagmin 1 (105103, Synaptic Systems, Göttingen, Germany) and a secondary antibody (dilution 1:100) fluorescently labelled with Atto 647N (40839, Sigma-Aldrich, St Louis, USA).

### CEST experiments

We employed CEST measurements^28^,^36^,^37^,^38^,^40^ to gain a deeper understanding of the equilibrium between membrane unbound and membrane-bound states of αS. In the study of αS–SUV interactions, CEST shows enhanced characteristics compared to standard heteronuclear correlation spectroscopy, including a significantly higher sensitivity at low lipid:protein ratios, conditions under which protein or lipid aggregation can be minimized^64^. The resulting NMR signals enable the interaction between αS and the membrane surface to be probed without interference from additional factors that may influence the transverse relaxation rates of the protein resonances^36^,^37^,^38^,^40^. In the CEST experiments employed here, a continuous weak radiofrequency field (either 350 or 170 Hz) was applied off-resonance (up to ±28 kHz) in the ^15^N channel, thereby saturating the broad spectroscopic transitions in the bound (undetectable) state but leaving the resonances of the free (detectable) state virtually unperturbed^36^,^37^,^38^,^40^. The saturation of the bound state was then transferred to the free state via chemical exchange, resulting in the attenuation of the intensities of the observable resonances in the visible unbound state. By carrying out a series of experiments at various offsets, it was possible to obtain a map of the strength of the interactions between the low (unbound αS) and high (SUV-bound αS) molecular weight species at a residue specific resolution.

Solution-state NMR experiments were carried out at 10 °C on Bruker spectrometers operating at ^1^H frequencies of 700 MHz equipped with triple resonance HCN cryo-probes. CEST experiments were based on ^1^H–^15^N HSQC experiments by applying constant wave saturation in the ^15^N channel. Since we aimed at probing the exchange between monomeric αS (having sharp resonances) and αS bound to SUVs (having significantly broader resonances), a series of large offsets was employed (−28, −21, −14, −9, −5, −3, −1.5, 0, 1.5, 3, 5, 9, 14, 21 and 28 kHz), resulting in CEST profiles of symmetrical shapes (Supplementary Fig. 1)^28^,^36^,^37^. An additional spectrum, saturated at −100 kHz, was recorded as a reference. The CEST experiments were recorded using a data matrix consisting of 2,048 (t_2_, ^1^H) × 220 (t_1_, ^15^N) complex points. Assignments of the resonances in ^1^H–^15^N HSQC spectra of αS_WT_ were derived from our previous studies^28^ whereas assignments of the mutational variants employed in this work were obtained by a standard combination of triple resonance solution NMR spectra^66^.

### MAS measurements

MAS provides complementary information to CEST as it allows the protein resonances in the vesicle-bound state, which is inaccessible to solution-state NMR, to be probed directly. MAS experiments were carried out on a 16.85 T Bruker Spectrometer with a 3.2 mm E^Free^ probe. DARR experiments^42^ were performed at a MAS rate of 10 kHz using a series of different mixing times (20, 50, 100, 200 and 500 ms), and the spectra were acquired at −19 and 4 °C (the latter for control experiments only) using a 1 ms contact time. INEPT spectra^41^ were measured at 4 °C using a MAS rate of 10 kHz. Pulse widths were 2.5 μs for ^1^H and 5.5 μs for ^13^C, and ^1^H TPPM decoupling was applied at ωRF/(2π)=71.4–100 kHz (ref. 28).

### Cryo-EM measurements

All samples used in cryo-EM measurements were incubated, with or without αS (200 μM), for 12 h at 298 K using fresh preparations of DOPE:DOPS:DOPC SUVs at a concentration of 0.05%. After incubation cryo-EM grids were prepared by vitrifying the sample solutions using aliquots of 2 μl and a Vitrobot Mark IV (FEI, Hillsboro, USA) at a relative humidity of 100%. The samples were loaded on a glow-discharged Quantifoil Copper 300 mesh R2/2 grids (Quantifoil Micro Tools GmbH, Germany) and blotted with filter paper for 2.5 s to leave a thin film of solution. The blotted samples were plunged into liquid ethane and stored under liquid nitrogen before imaging. Samples were examined using a Philips CM200 FEG electron microscope operating at 200 kV (FEI, Hillsboro, USA), using a Gatan 626 cryo-holder (Gatan, Pleasantos, USA) cooled with liquid nitrogen to temperatures below −180 °C. Digital images were acquired on a TVIPS FC415 CCD camera using the EMMENU 4 software package (TVIPS, Munich, Germany).

### STED microscopy

STED imaging^67^,^68^ was carried out by fluorescently labelling the DOPE:DOPS:DOPC SUVs with 2% of fluorescently labelled DOPE (ATTO 647N DOPE, ATTO-TECH, USA). STED microscopy allows the diffraction limit in optical microscopy (∼200 nm) to be overcome^69^ and imaging was performed on a home-built pulsed STED microscope^69^ using a single titanium-sapphire oscillator centred at *λ*_STED_=765 nm (Ti:S, Mai Tai HP, Spectra-physics, Santa Clara, USA) to generate the STED beam, which was subsequently split into two using a half-plate and a polarization beam splitter. Of these two beams, the one transmitted was focused onto a photonic crystal fibre (FemtoWhite, NKT Photonics, Cologne, Germany) to produce white light radiation. From this light, an excitation beam, centred at *λ*_Exc_=640 nm, was extracted using a bandpass filter (637/7 BrightLine HC, Semrock, NY, USA) and coupled into a 30 m long polarization maintaining single-mode fibre (PM630-HP, Thorlabs, Newton, UK). The reflected STED beam was passed through a 50 cm long glass block of SF66 and a 100 m long polarization maintaining single-mode fibre (PM-S630-HP, Thorlabs, Newton, UK) to stretch the pulse duration to ∼100–200 ps. In addition, the STED beam was converted into a so called donut beam by a spatial light modulator (X10468−02, Hamamatsu Photonics, Hamamatsu City, Japan). The excitation and STED beams were recombined with a dichroic mirror (T735spxr, Chroma, Bellow Falls, USA) and detected using a commercial point-scanning microscope (Abberior Instruments, Göttingen, Germany) comprising of a microscope frame (IX83, Olympus, Shinjiuku, Japan), a set of galvanometer mirrors (Quad scanner, Abberior Instruments, Göttingen,, Germany) and a detection unit. The beams were focused onto the sample by a × 100/1.4 NA oil immersion objective lens (UPLSAPO 100XO, Olympus, Göttingen, Germany) and images were acquired by raster scanning the beams across the sample using the Inspector software (Andreas Schönle, Max Planck Institute for Biophysical Chemistry, Göttingen, Germany). We used a field of view of 30 × 30 μm^2^ with a pixel size of 15 × 15 nm^2^ and a pixel dwell time of 20 μs. Fluorescence photons emerging from the sample were collected by the microscope objective lens, de-scanned by the galvanometer mirrors, focused onto a pinhole and detected using an avalanche photodiode (SPCM-AQRH, Excelitas Technologies. Waltham, USA). The laser powers, measured at the objective back aperture, were ca. 20 μW and 150 mW for the excitation beam and for the STED beam, respectively.

### Analysis of STED images for vesicle size measurement

Vesicle sizes were estimated from STED images by using in-house Matlab scripts (Supplementary data 1). First, images of isolated vesicles were identified and analysed using a fitting based on a 2D Gaussian function, by convolving the images with a Gaussian filter whose dimensions match the extension of the expected STED point spread function. The centres of the vesicles were identified by finding local maxima of the convolved images, excluding the local maxima corresponding to fused vesicles by means of a threshold applied on the peak intensities. A different fitting procedure was optimized in the case of assembled vesicles that appear as hollow shapes in the STED images, as for a vesicle larger than the lateral resolution of the STED microscope the number of dye molecules probed increases on the edge of the shell. To estimate the size of the clusters and their relative number compared to the non-fused vesicles, all the vesicles appearing as fused were fitted by annular functions having a Gaussian radial profile (amplitude, position, radius and offset) using a nonlinear least squares approach.

### Direct stochastic optical reconstruction microscopy

Super-resolution imaging was performed using dSTORM microscopy with a Nikon Eclipse TE 300 inverted wide-field microscope using a × 100, 1.49-N.A total internal reflection fluorescence^46^ objective lens (Nikon Ltd., Kingston upon Thames, UK). The vesicle and αS samples were adhered to a glass coverslip coated in poly-L-lysine (P4707, Sigma-Aldrich, St Louis, USA) before photoswitching buffer solution was added, consisting of 100 mM mercaptoethylamine (MEA) in phosphate buffered saline (PBS, pH 8.2). For imaging, a laser emitting at a wavelength of 640 nm was used (Toptica Photonics AG, Graefelfing, Germany) for excitation of the Atto 647N dye. A 405 nm laser (Mitsubishi S3 Electronics Corp., Tokyo, Japan) was used as the reactivation source, which was only turned on when the number of active fluorophores in the field of view was visibly reduced. Imaging was performed under total internal reflection fluorescence illumination conditions, ensuring that the exact centre of the field of view was illuminated. The field of view covered 1,997 × 1,997 camera pixels, corresponding to an area on the sample of ∼20 × 20 μm^2^. 10,000 fluorescence frames were recorded, each corresponding to an exposure time of 10 ms; the latter was matched to be in the range of the average ‘on' time of the fluorescent dyes. The fluorescence light in the detection path was filtered and imaged with an Ixon DV887 ECS-BV EM-CCD camera (Andor, Belfast, UK). The image analysis was performed using frames 1000 to 10 000 in each sequence. From each image stack, a reconstructed *d*STORM image was generated using the open-source rapidSTORM software (Supplementary Data 2) developed in house using MATLAB (The MathWorks, Inc.).

### Dynamic light scatterning

DLS measurements of vesicle size distributions were performed using a Zetasizer Nano ZSP instrument (Malvern Instruments, Malvern, UK) with backscatter detection at a scattering angle of 173°. The viscosity (0.8882 cP) and the refractive index (1.330) of water were used as parameters for the buffer solution, and the material properties of the analyte were set to those of the lipids (absorption coefficient of 0.001 and refractive index of 1.440). SUVs were used at a concentration of 0.05% in these measurements and the experiments were performed at 25 °C. The acquisition time for the collection of each dataset was 10 s and accumulation of the correlation curves was obtained using 10 repetitions. Each measurement was repeated 10 times to estimate standard deviations and average values of the centres of the size distributions (Supplementary Fig. 11).

### Modelling

Schematic representations of αS bound to SUVs were obtained by using molecular dynamics (MD) simulations in implicit solvent. The structure of αS in the double-anchor mechanism (Fig. 3a) were obtained by starting from the model of membrane-bound conformation αS characterized by an elongated helix (residues 1 to 97) with a disordered C-terminal region (residues 98–140) which was part of the ensemble characterized previously^28^. Atomic coordinates (in Cartesian space) of the N-terminal anchor were harmonically restrained to maintain a fixed position whereas the region spanning residues 65–97 was restrained in the alpha-helical conformation. A constant force along the membrane normal was applied to this region to extend it toward the second vesicle (up in the Fig. 3a). The reminder of the protein (residues 26–59 and 98–140) was allowed to relax under the Newtonian laws of motions during the MD simulations. Curved vesicle surfaces were generated by starting from atomic models of DOPE:DOPS:DOPC bilayers and by generating roto-translations that imposed a spherical symmetry with a radius of 25 nm.

### Data availability

Data supporting the findings of this study are available within the article and its Supplementary Information Files and from the corresponding author on request.

## Additional information

**How to cite this article:** Fusco, G. *et al*. Structural basis of synaptic vesicle assembly promoted by α-synuclein. *Nat. Commun.* 7:12563 doi: 10.1038/ncomms12563 (2016).

## Supplementary Material

Supplementary Information Supplementary Figures 1-11

Supplementary Data 1 Matlab scripts for the analysis of super-resolution images.

Supplementary Data 2 Instructions for obtaining rapidSTORMM

## Figures and Tables

**Table 1:** Primers used in this study.

**Primer Name**	**Variant**	**Sequence**	**Tm (°C)**
K80E_F	αS_Sw_	5′- AGTAGCCCAGGAGACAGTGGAG -3′	65
K80E_R*	αS_Sw_	5′- GCTGTCACACCCGTCACC -3′	66
A30P_F	αS_A30P_	5′- GGCAGAAGCACCTGGAAAGACAAAAG -3′	56
A30P_R*	αS_A30P_	5′- ACACCCTGTTTGGTTTTC -3′	57
E46K_F	αS_E46K_, αS_Sw_	5′- CAAAACCAAGAAGGGAGTGGTG -3′	60
E46K_R*	αS_E46K_, αS_Sw_	5′- GAGCCTACATAGAGAACAC -3′	57

^*^Reverse primer.
