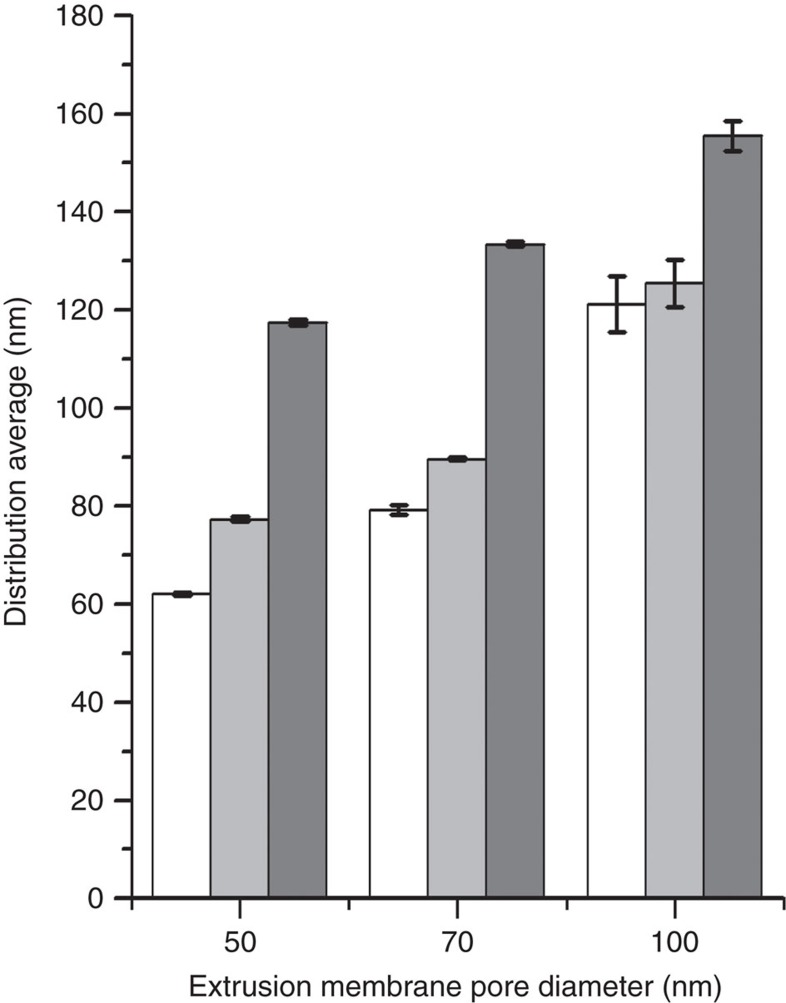# Corrigendum: Structural basis of synaptic vesicle assembly promoted by α-synuclein

**DOI:** 10.1038/ncomms15667

**Published:** 2017-05-11

**Authors:** Giuliana Fusco, Tillmann Pape, Amberley D. Stephens, Pierre Mahou, Ana Rita Costa, Clemens F. Kaminski, Gabriele S. Kaminski Schierle, Michele Vendruscolo, Gianluigi Veglia, Christopher M. Dobson, Alfonso De Simone

Nature Communications 7: Article number: 12563; DOI: 10.1038/ncomms12563 (2016); Published: 09 19 2016; Updated 05 11 2017

In the original version of Supplementary Data 1 associated with this Article, the FastPeakFind.m function was incorrectly attributed to the authors in the function's header. This function is Copyright © 2012 Adi Natan, Stanford University (natan@stanford.edu). The HTML version of the Article has been updated to include a revised Supplementary Data 1 with the correct attribution.

In the Supplementary Information file, there are errors in the labelling of the *x* axis in Supplementary Fig. 11. The *x* axis label should be ‘Extrusion Membrane Pore Diameter (nm)' and the labels ‘SUV', ‘WT' and ‘E46K/K80E' should be ‘50', ‘70' and ‘100', respectively. The correct version of this figure appears as Fig. 1 below. [Fig f1]

## Figures and Tables

**Figure 1 f1:**