# Supplementary material for: Structural basis of synaptic vesicle assembly promoted by α-synuclein
Source: Nat Commun. 2016 Sep 19;7:12563. doi: 10.1038/ncomms12563 (PMC5031799; doi:10.1038/ncomms12563)
Supplement: Supplementary Information — Supplementary Figures 1-11 [file ncomms12563-s1.pdf]

Supplementary Figures

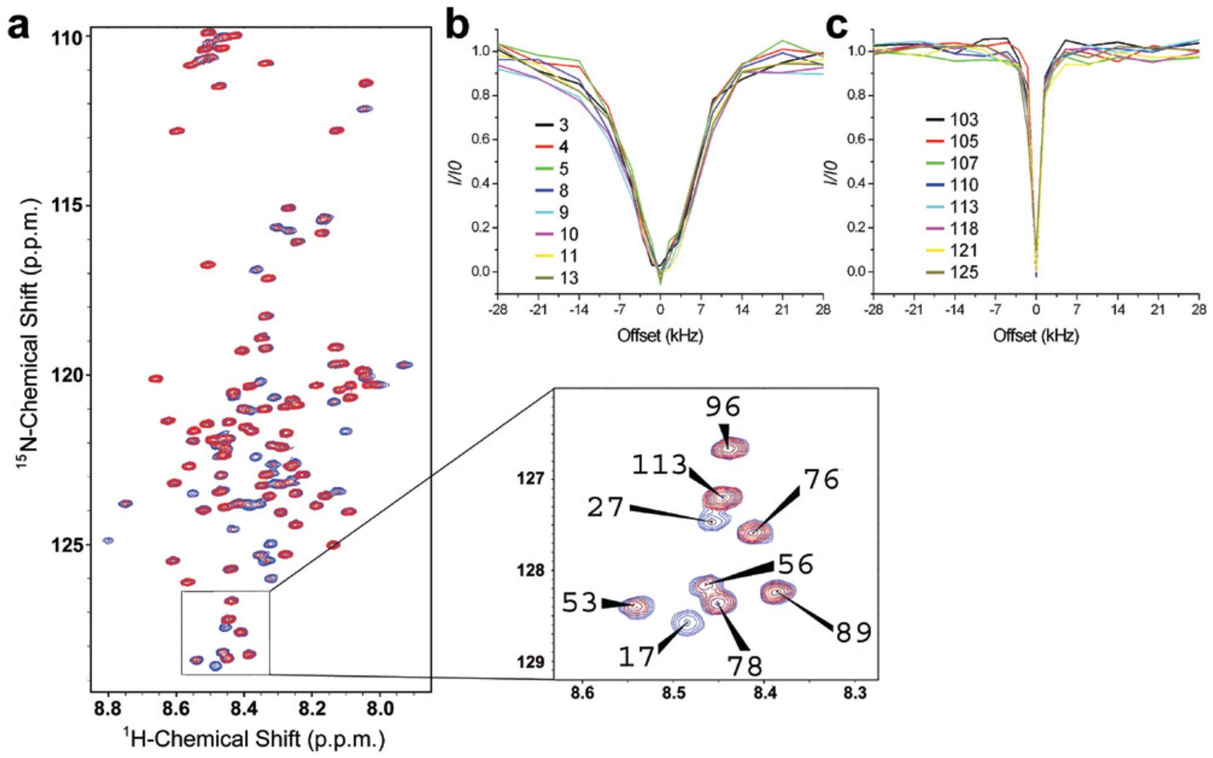

Supplementary Figure 1 | CEST experiment. (**a**) Two representative CEST spectra based ^{1}H-^{15}N-HSQC of αS_{WT}. Blue and red spectra represent CEST ^{1}H-^{15}N-HSQC measured using a 350 Hz continuous wave length at offsets of 100 kHz (reference) and 1.5 kHz, respectively. (**b-c**) Individual CEST profiles for residues at the N- and C-termini are shown in panels b and c, respectively. These CEST experiments were recorded at a ^{1}H frequency of 700 MHz using a protein concentration of 300 μM and 0.06% (0.6 mg ml^{-1}) of DOPE:DOPS:DOPC lipids in a ratio of 5:3:2 and assembled in SUVs. ^{1}H-^{15}N HSQC spectra were recorded by using a continuous wave saturation (170 Hz or 350 Hz) on the ^{15}N channel at offsets ranging between -28 kHz and +28 kHz. An additional spectrum, saturated at -100 kHz was recorded as a reference. Data recorded using 350 Hz are shown.

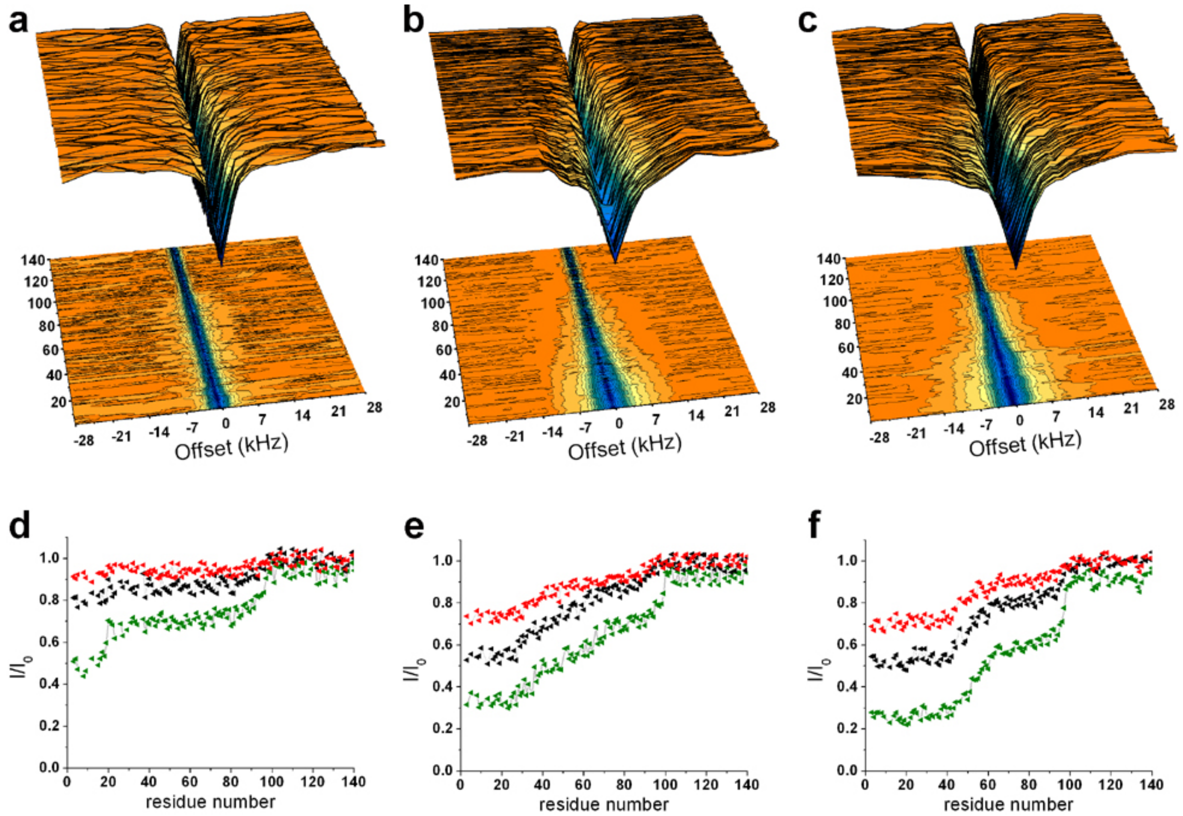

Supplementary Figure 2 | CEST experiments of αS_{A30P} and αS_{E46K} using a continuous wavelength of 170Hz. ^{1}H-^{15}N HSQC spectra recorded by using a continuous wave saturation of 170 Hz in the ^{15}N channel at offsets ranging between -28 kHz and +28 kHz. An additional spectrum, saturated at -100 kHz was recorded as a reference. For the sake of comparison, plots in panels ***b*** and ***e*** are drawn using αS_{WT} data from our previous investigation[27]. (**a-c**) CEST surfaces for αS_{A30P} (a) αS_{WT}[27] (b) and αS_{E46K} (c). (**d-f**) CEST saturation along the sequences of αS_{A30P} (d) αS_{WT}[27] (e) and αS_{E46K} (f). Green lines refer to the averaged CEST profiles measured using offsets at +/- 1.5 kHz. Similarly, profiles for +/- 3 kHz and +/- 5 kHz are shown in red and black, respectively.

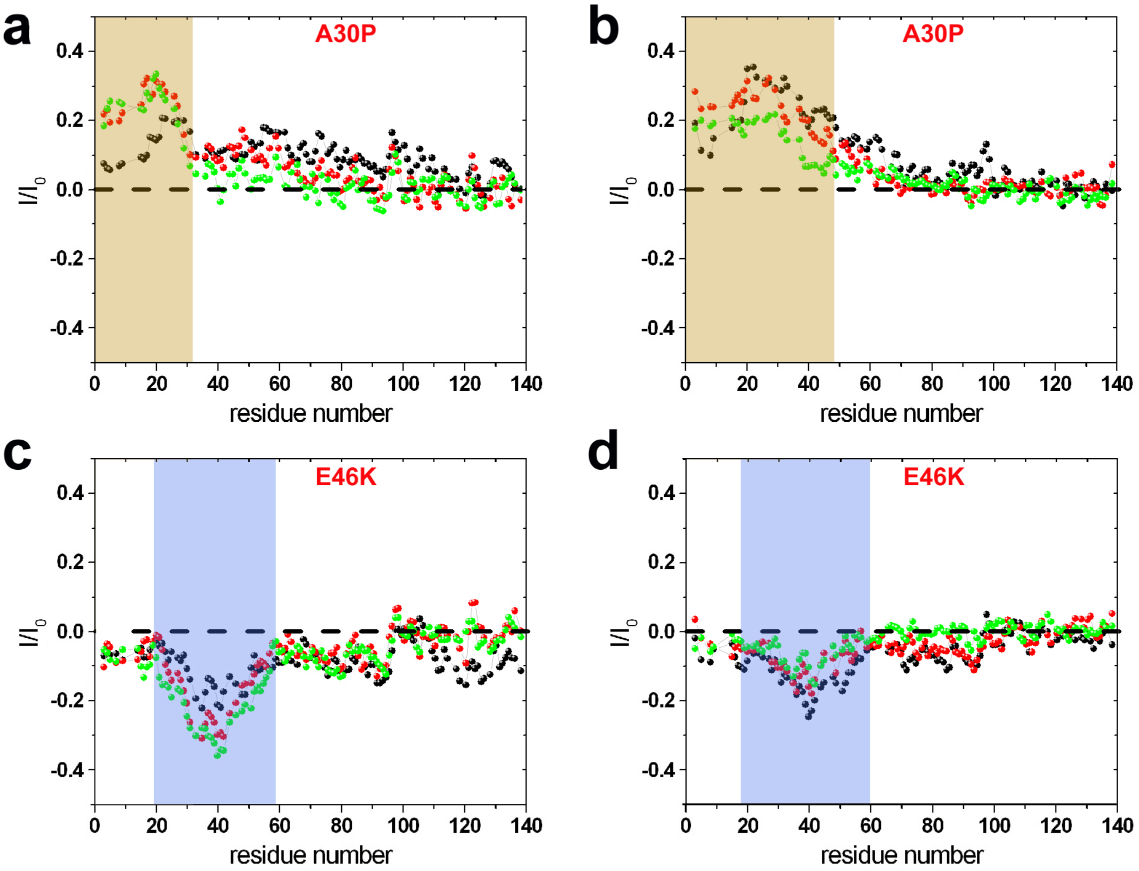

Supplementary Figure 3 | CEST differences between αS$_{WT}$ and αS$_{A30P}$ and αS$_{E46K}$. CEST profiles of the αS$_{WT}$ variants have been subtracted from those of αS$_{A30P}$ and αS$_{E46K}$. Green, red and black lines are used for offsets of +/- 1.5 kHz, +/- 3.0 kHz and +/- 5.0 kHz, respectively. (**a-b**) Difference in CEST profiles of αS$_{A30P}$ and αS$_{WT}$, measured with bandwidths of 350Hz (**a**) and 170Hz (**b**). The three offsets show a peak of positive differences in the regions indicated in yellow, showing that in this region the saturation is weaker for αS$_{A30P}$ than for αS$_{WT}$. (**c-d**) Difference in CEST profiles of αS$_{E46K}$ and αS$_{WT}$, measured with bandwiths of 350Hz (**c**) and 170Hz (**d**). Green, red and black lines are used for offsets of +/- 1.5 kHz, +/- 3.0 kHz and +/- 5.0 kHz, respectively. The three offsets show a peak of negative differences in the regions indicated in blue, showing that in this region the saturation is stronger for αS$_{E46K}$ than for αS$_{WT}$.

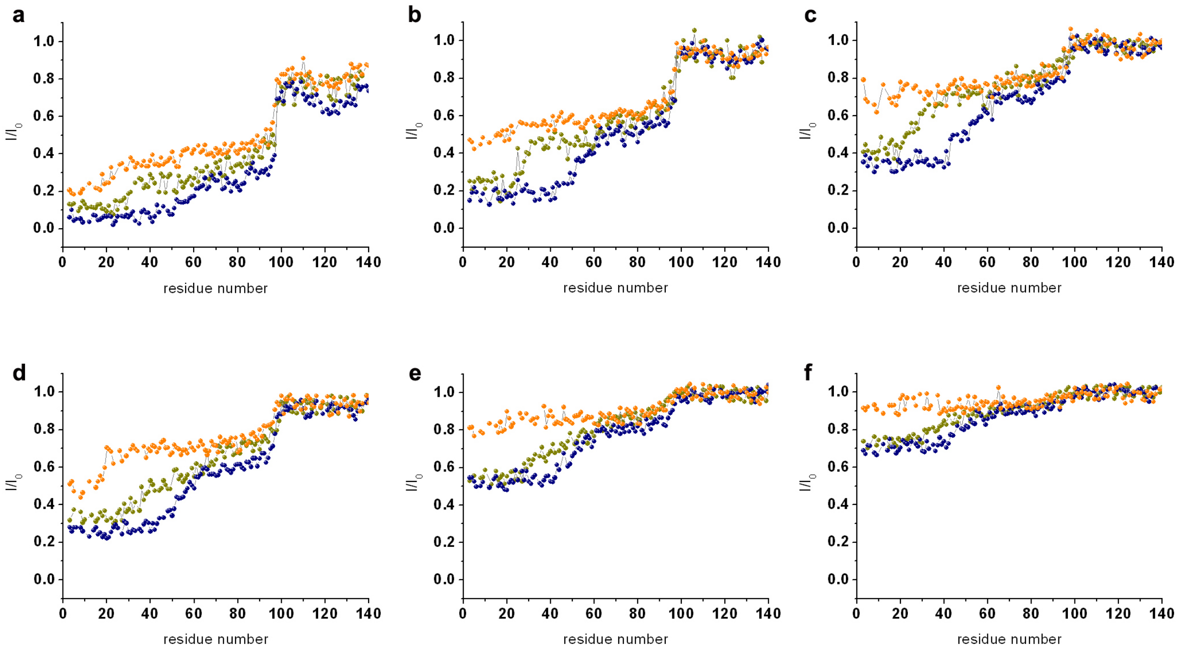

Supplementary Figure 4 | CEST profiles of αS$_{WT}$ and αS$_{A30P}$ and αS$_{E46K}$ at different offsets. Color codes are green, orange and blue for αS$_{WT}$, αS$_{A30P}$ and αS$_{E46K}$, respectively. Panels a, b and c report measurements performed with a saturation bandwidth of 350Hz and using offsets of +/- 1.5 kHz, +/- 3.0 kHz and +/- 5.0 kHz, respectively. Panels d, e and f report measurements performed with a saturation bandwidth of 170Hz and using offsets of +/- 1.5 kHz, +/- 3.0 kHz and +/- 5.0 kHz, respectively. The CEST profiles of these αS variants reveal a degree of independence between the membrane binding properties of the N-terminal region (membrane-anchor), whose membrane-binding properties are significantly affected in αS$_{A30P}$ and αS$_{E46K}$, and of the region spanning residues 65 to 97, which is mainly unaffected by the A30P and E46K mutations. This trend is particularly defined when large offsets for the saturation are used in CEST (panels b, c, e and f). Under these conditions, CEST is sensitive mainly to the saturation transfer associated with αS conformations that are tightly bound with the vesicle surface, as the NMR peaks corresponding to these states feature linewidths of several kHz. Conversely, at small offsets (± 1.5 kHz) CEST profiles rely on both tightly bound states and tethered conformations that are aspecifically adsorbed onto the lipid bilayer, as the latter feature less broad peaks than in the case of tightly bound conformations. This is exemplified in the case of the transient interactions between the C-terminal region of αS and the membrane surface, which result in a mild saturation transfer when CEST is measured using a bandwidth of 350 Hz at ± 1.5 kHz (panel a), suggesting that tethered conformations on the membrane surface are transiently populated for this region of the protein, as also probed by PRE experiments (Supplementary Figure 5).

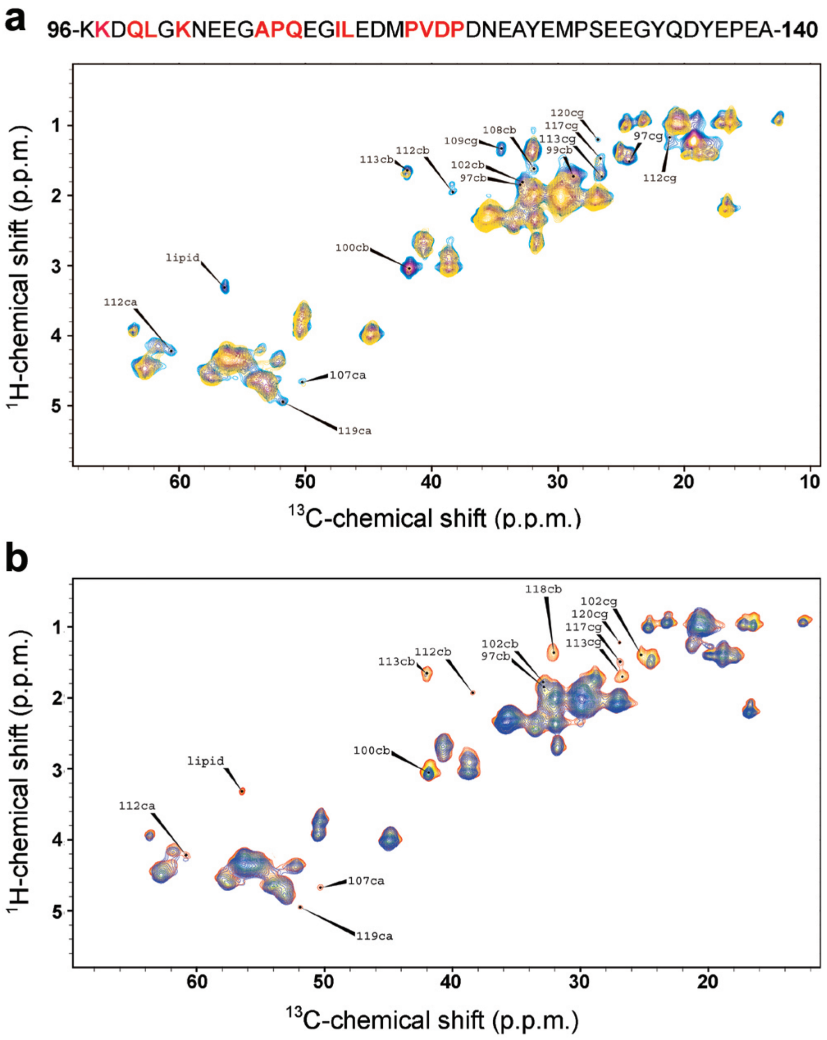

Supplementary Figure 5 | Paramagnetic relaxation experiments. αS_{A30P} and αS_{E46K} PRE data with paramagnetic SUVs are shown in panels a and b, respectively. To obtain paramagnetic vesicles, the DOPE:DOPS:DOPC mixture was doped with 2% of (1,2-dimyristoyl-sn-glycero-3-phosphoethanolamine-N-DTPA, gadolinium salt). (**a**) Comparison between INEPT spectra of αS_{A30P} with (yellow) and without (blue) the spin label. (**b**) Comparison between INEPT spectra of αS_{E46K} with (blue) and without (yellow) the spin label. The two variants show similar patterns of enhanced relaxation, which indicated intermolecular contacts due to the spatial proximity of the atoms of αS and the unpaired spins on the surfaces of the SUVs. Residues that showed PRE effects with the membrane are found to be mainly hydrophobic and have been indicated in red in the sequence of the disordered C-terminal tail (top of panel **a**).

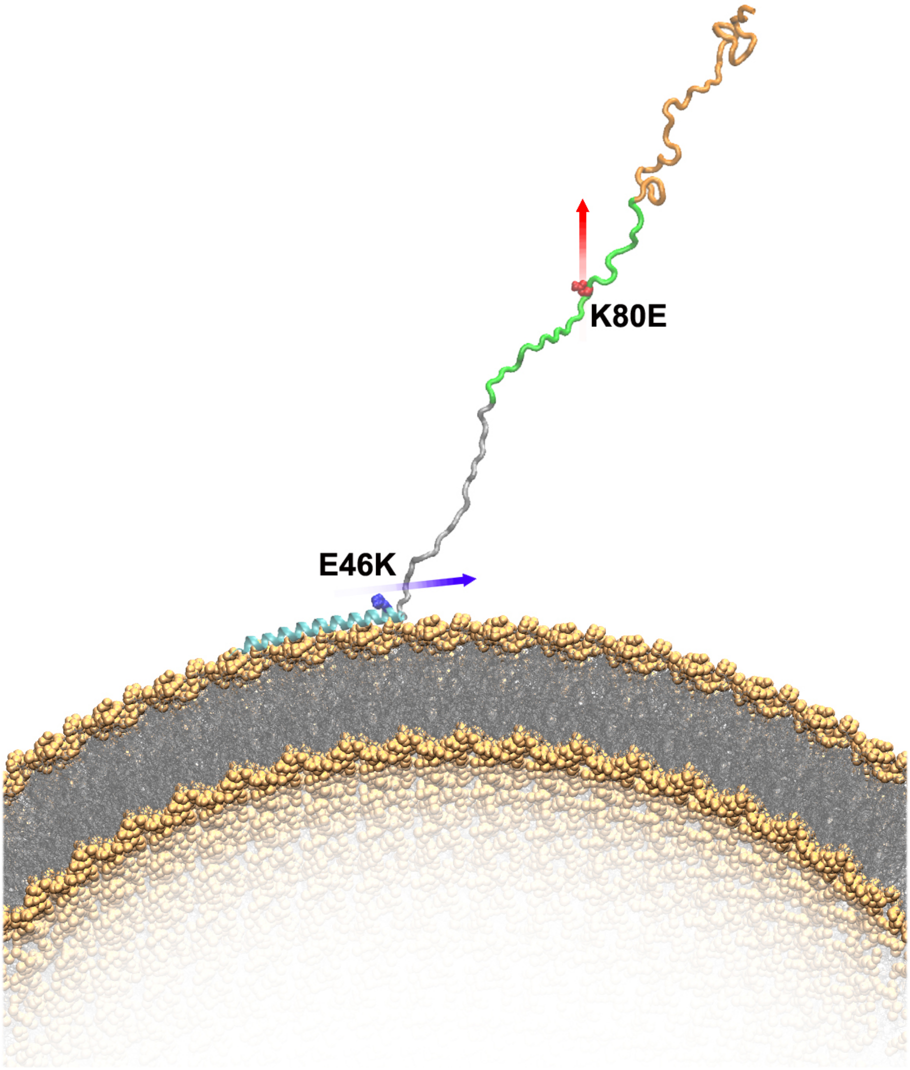

Supplementary Figure 6 | Schematic illustration of the membrane binding properties of αS$_{Sw}$. The swapped mutant of α-synuclein (αS$_{Sw}$) described in this study incorporates two specific point mutations. The K80E mutation, by disfavouring the local binding to negatively charged lipid bilayers, enhances the population of the conformational states of αS that feature the detachment of the region 65 to 97 from the surface of SUVs. In addition, the second mutation (E46K) strengthens the binding of the N-terminal anchor region, which according to our solution-state and solid-state NMR data is extended to residue 42, thereby restoring an overall K_D comparable to that of the wild type protein. In this schematic illustration of the membrane bound state of αS$_{Sw}$, a conformation featuring the detachment of the region 65 to 97 of αS from the surface of SUVs is drawn. Color codes for the different regions are cyan, grey, green and orange for the regions 1 to 42, 43 to 64, 65 to 97 and 98 to 140, respectively. Residues K46 and E80 of αS$_{Sw}$ are drawn in blue and red, respectively. The effects of K46 in extending the membrane anchor region, and of E80 in detaching the region 65 to 97, are marked with blue and red arrows, respectively.

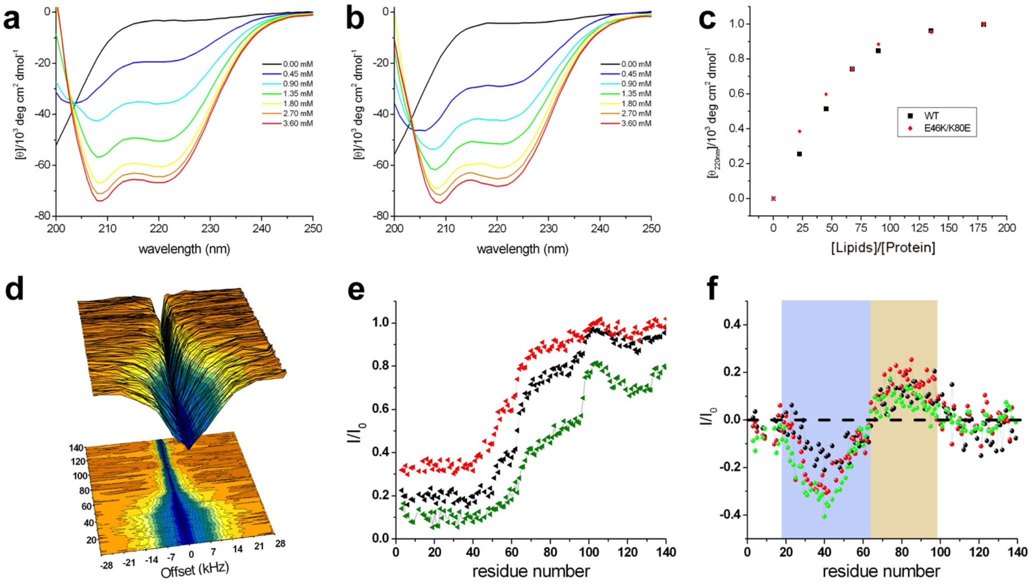

Supplementary Figure 7 | Differences in the structure, dynamics and binding affinity of αS_{Sw} and αS_{WT}. (a-b) CD analysis of αS_{WT} (**a**) and αS_{Sw} (**b**) in the presence of different concentrations of SUVs. In all measurements, the concentration of αS was kept constant at 20 μM. (**c**) Using the values of the ellipticity at 222 nm we estimated an apparent dissociation constant K_D of 207 ± 17 μM for αS_{Sw} and 261 ± 21 μM for αS_{WT} under the conditions employed in the present study. (**d**) CEST surface of αS_{Sw}. ^{1}H-^{15}N HSQC spectra were recorded by using a continuous wave saturation (350 Hz) on the ^{15}N channel at offsets ranging between -28 kHz and +28 kHz. An additional spectrum, saturated at -100 kHz was recorded as a reference. (**e**) CEST saturation along the sequences of αS_{Sw}. Green lines refer to the averaged CEST profiles measured using offsets of +/- 1.5 kHz. Similarly, profiles for +/- 3 kHz and +/- 5 kHz are shown in red and black, respectively. (**f**) CEST difference between αS_{WT} and αS_{Sw}. Green, red and black lines are used for offsets of +/- 1.5 kHz, +/- 3 kHz and +/- 5 kHz, respectively.

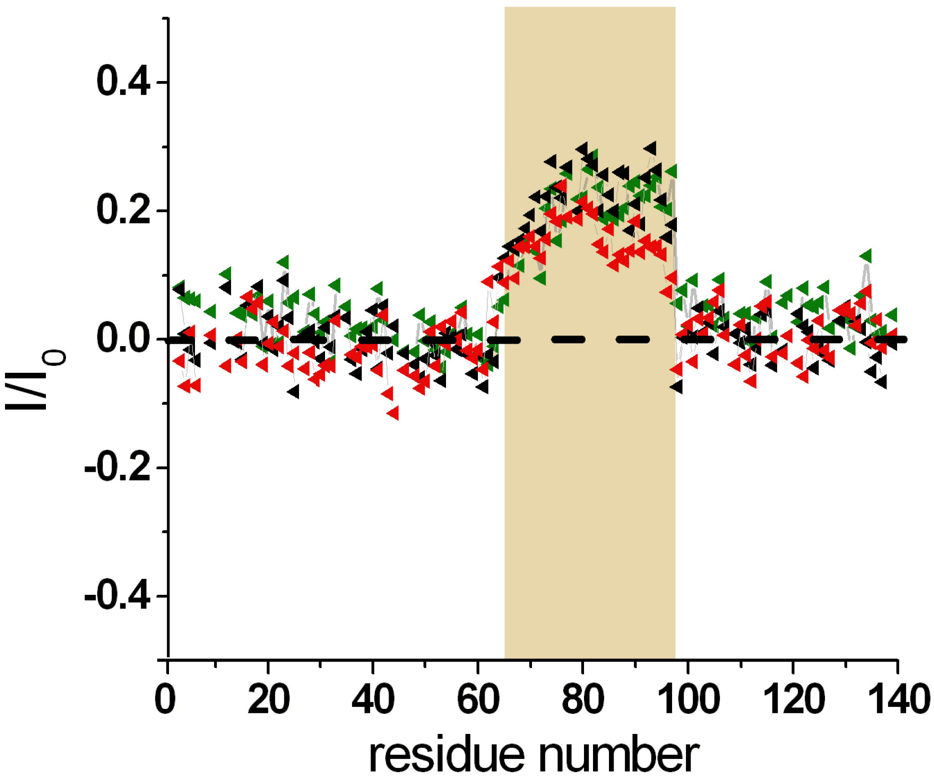

Supplementary Figure 8 | Differences in CEST profiles of αS$_{Sw}$ and αS$_{E46K}$. CEST profiles (with a bandwidth of 350Hz) of the αS$_{E46K}$ variants have been subtracted from those of αS$_{Sw}$. Green, red and black lines are used for offsets of +/- 1.5 kHz, +/- 3 kHz and +/- 5 kHz, respectively. The three offsets show a peak of positive difference in the regions indicated in yellow, showing that in this region saturation of αS$_{Sw}$ weaker than that of αS$_{E46K}$.

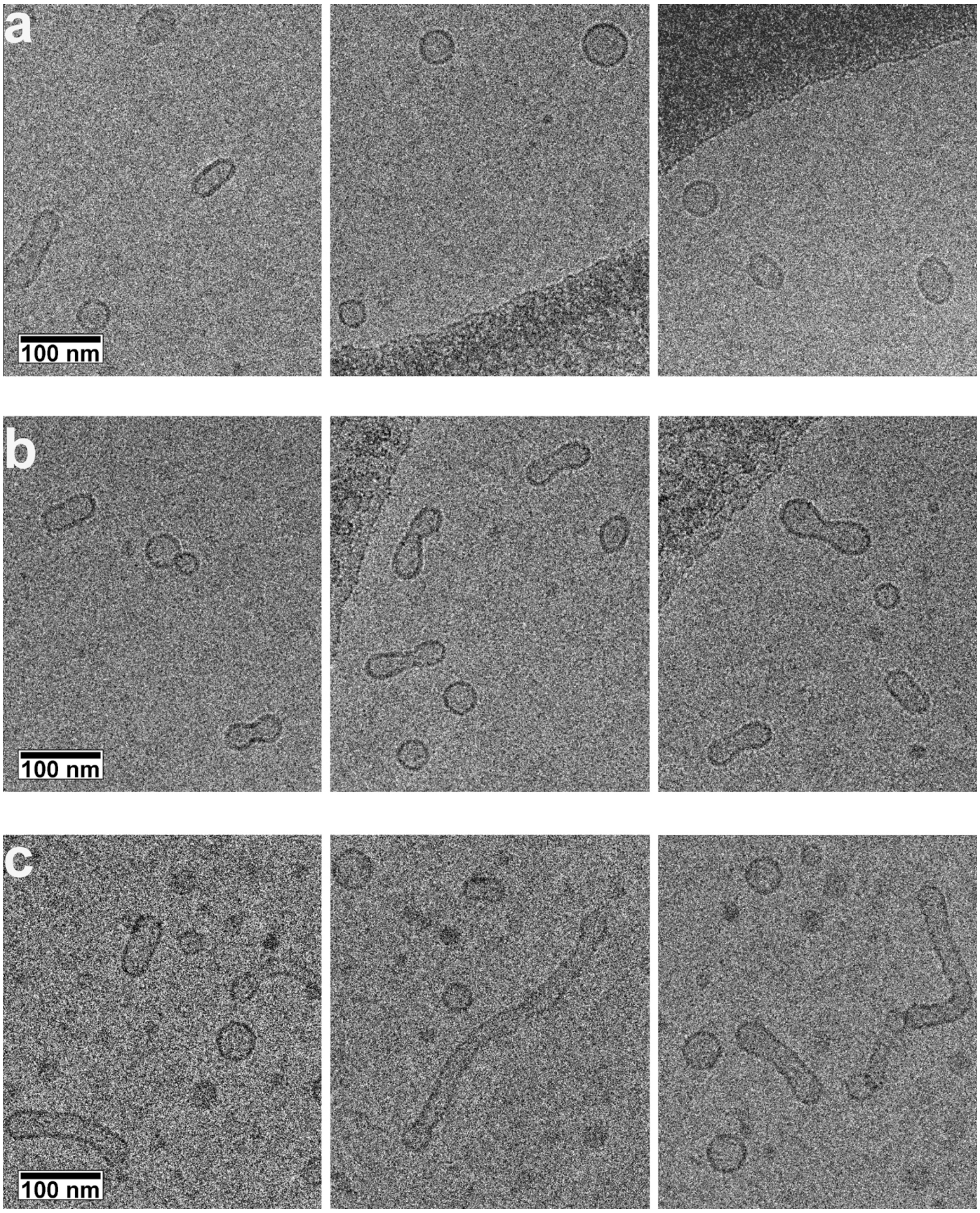

Supplementary Figure 9 | Gallery of cryo-EM images of SUVs in the presence and absence of αS$_{WT}$ and αS$_{Sw}$. (a) isolated SUV, (**b**) vesicles incubated with αS$_{WT}$, (**c**) vesicles incubated with αS$_{Sw}$. Samples used in cryo-EM measurements were incubated, with or without αS molecules (wild type and scrambled variants) at 200 μM concentration, for 12 h at 298 K using freshly prepared samples of 0.05% DOPE:DOPS:DOPC SUVs. After incubation, cryo-EM grids were prepared by vitrifying the sample solutions into liquid ethane and stored under liquid nitrogen prior to imaging. Samples were examined using a Philips CM200 FEG electron microscope operating at 200 kV (Philips, Amsterdam, NL), using a Gatan 626 cryo-holder (Gatan, Pleasantos, USA) cooled with liquid nitrogen to temperatures below −180 °C. Digital images were acquired on a TVIPS FC415 CCD camera with the EMMENU 4 software package (TVIPS, Munich, Germany).

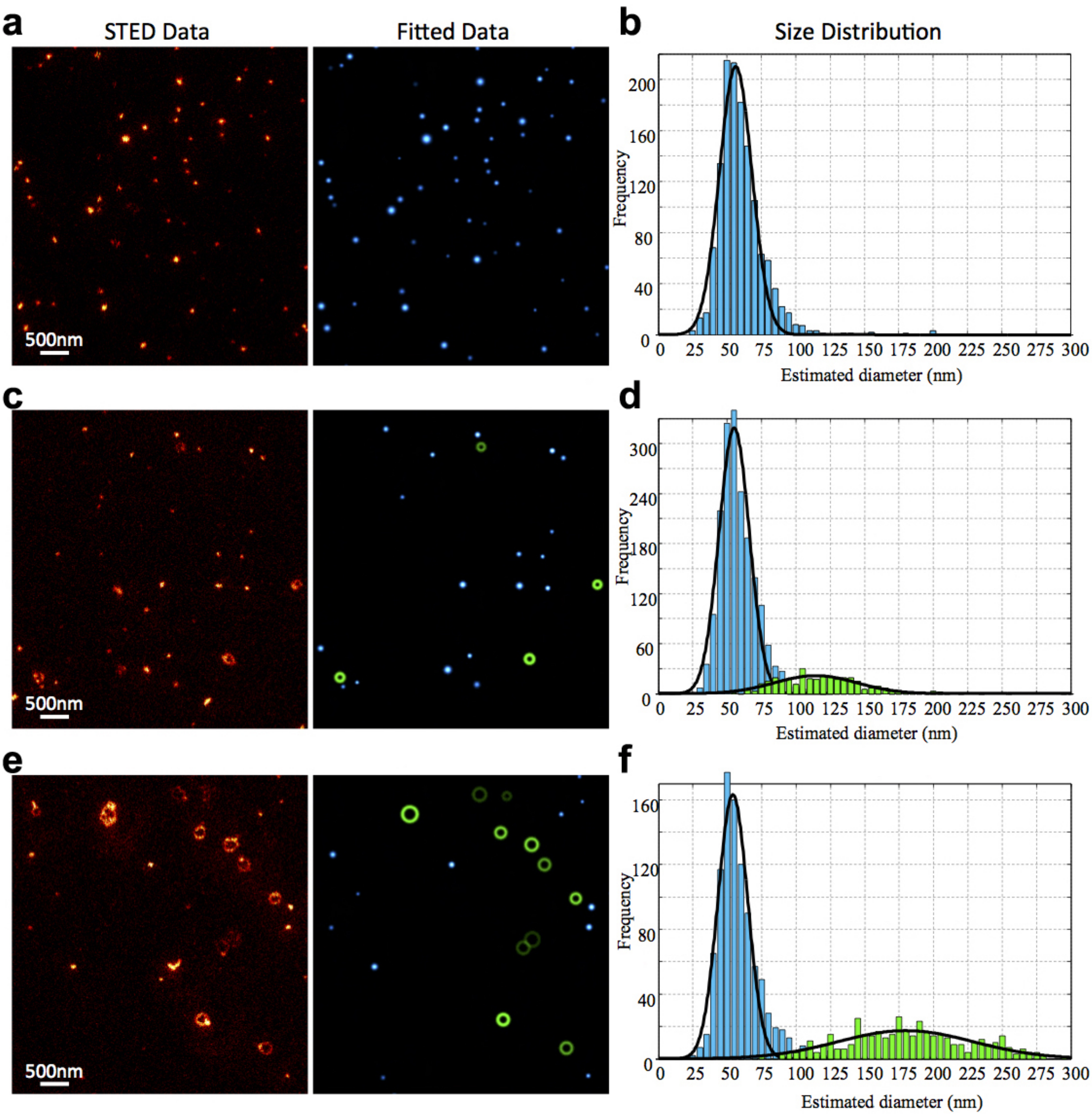

Supplementary Figure 10 | *Fitting models employed in STED images.* (**a**) STED image and (**b**) size distribution of ATTO labelled SUVs as isolated. 2D Gaussian fitting was employed to fit isolated vesicles (blue beads). (**c**) STED image and (**d**) size distributions of SUVs upon incubation with $\alpha S_{WT.}$ 2D Gaussian fitting (blue beads) and annular fitting (green rings) were employed to fit isolated and fused vesicles, respectively. (**e**) STED image and (**f**) size distributions of SUVs upon incubation with αS_{Sw}.

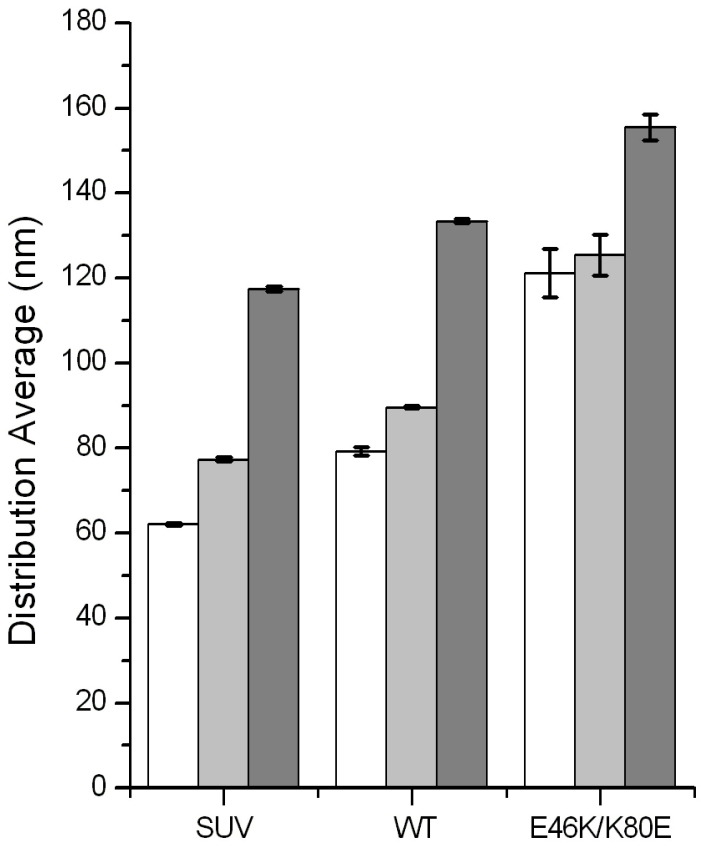

Supplementary Figure 11 | Average sizes of SUVs from DLS measurements. White bars represent the initial SUVs, whereas light gray and dark grey bars represent SUVs incubated with αS_{WT} and αS_{Sw}, respectively. Three different sizes were obtained by extrusion of the vesicles through membranes with different pore diameters. Regardless of the initial size, the incubation of the vesicles with αS_{WT} induces an increase of the average size of the DLS distribution. A stronger effect, however, is measured upon incubation with αS_{Sw}. In the latter case, the measurements were associated with larger standard deviations; this finding is probably associated with the bias of DLS arising from the enhanced scattering properties of large particles. Despite these biases, the measurements consistently showed an increase in size in the αS_{Sw}-incubated samples. Thus, while the enhanced scattering properties of large particles affect the average values of the distributions, the DLS measurements conclusively show an enhanced ability of αS_{Sw} to favour interaction and fusion of synaptic-like vesicles, which is in agreement with STED and cryo-EM measurements.

Each measurement was made of 10 replicates. Error bars report standard deviations of the centres of the size distributions in the 10 replicates.
