# Supplementary material for: Structural basis of synaptic vesicle assembly promoted by α-synuclein
Source: Nat Commun. 2016 Sep 19;7:12563. doi: 10.1038/ncomms12563 (PMC5031799; doi:10.1038/ncomms12563)
Supplement: Supplementary Data 1 — Matlab scripts for the analysis of super-resolution images. [file ncomms12563-s2.pdf]

Supplementary Data 1. STED imaging code.

Readme

Pierre Mahou // University of Cambridge //
pierre.mahou@polytechnique.edu

```
Content:
        -Readme
        -Licence for Matlab function FastPeakFind
        -Matlab script FastPeakFind
        -License for remaining Matlab functions
        -Matlab scripts:
                -Fit_Gaussian
                -Fit_One_Bead_Gauss
                -Fit_One_Ring_Gauss
                -Main_Affine
                -Ring_Finder_Affine
--------------------------------------------------------------------------------------

Unfused vesicles are processed by the Matalb script: Main_Affine

How the code works:
%%% Spherical objects in STED data are identified and their positions/widths
%%% are fitted. Objects too dim or too bright can be removed
%%% to exclude artefactual objects in the images. Objects too close to each other can
    also be removed
%%% as they tend to bias the results of the fitting.
%%% The fitting function is a 2D gaussian
%%% If Mask_Init = 0, the objects are identified as described above and only the good
%%% candidates are fitted. This strategy can be used to refine gradually
%%% the objects to fit by editing the Guess image i.e. by removing the false positives
    and adding
%%% the approximate positions of the "forgotten objects".
%%% Editing can be done for e.g. with Fiji. All the fitted positions
%%% at the end of the loop are stored in the image: Rings-Guess-Out-Pos
%%% Results of the fits can be evaluated by inspecting the image: Vesicles-Guess-Out

----------------------------------------------------------------------------------------

Fused vesicles are processed by the Matlab script: Ring_Finder_Affine

How the code works:
%%% Annular objects in STED data are identified and their positions/widths are fitted.
%%% The fitting function is a annulus with a Gaussian radial profile
%%% If Mask_Init = 1, the script loads an image where a Guess of the objects positions
    are stored (Img_Ring_Pos).
%%% In this case only the objects stored in the Guess are fitted
%%% If Mask_Init = 0, the objects are identified as described above and only the good
%%% candidates are fitted. This strategy can be used to refine gradually
%%% the objects to fit by editing the Guess image i.e. by removing the false positives
    and adding
%%% the approximate positions of the "forgotten objects".
%%% Editing can be done for e.g. with Fiji. All the fitted positions
%%% at the end of the loop are stored in the image: Rings-Guess-Out-Pos
%%% Results of the fits can be evaluated by inspecting the image: Rings-Out-Fit
```

Licence for FastPeakFind.m

Copyright © 2012, Adi Natan, Stanford University (natan@stanford.edu)
All rights reserved.

Redistribution and use in source and binary forms, with or without modification, are permitted provided that the following conditions are met:

* Redistributions of source code must retain the above copyright notice, this list of conditions and the following disclaimer.
* Redistributions in binary form must reproduce the above copyright notice, this list of conditions and the following disclaimer in the documentation and/or other materials provided with the distribution

THIS SOFTWARE IS PROVIDED BY THE COPYRIGHT HOLDERS AND CONTRIBUTORS "AS IS" AND ANY EXPRESS OR IMPLIED WARRANTIES, INCLUDING, BUT NOT LIMITED TO, THE IMPLIED WARRANTIES OF MERCHANTABILITY AND FITNESS FOR A PARTICULAR PURPOSE ARE DISCLAIMED. IN NO EVENT SHALL THE COPYRIGHT OWNER OR CONTRIBUTORS BE LIABLE FOR ANY DIRECT, INDIRECT, INCIDENTAL, SPECIAL, EXEMPLARY, OR CONSEQUENTIAL DAMAGES (INCLUDING, BUT NOT LIMITED TO, PROCUREMENT OF SUBSTITUTE GOODS OR SERVICES; LOSS OF USE, DATA, OR PROFITS; OR BUSINESS INTERRUPTION) HOWEVER CAUSED AND ON ANY THEORY OF LIABILITY, WHETHER IN CONTRACT, STRICT LIABILITY, OR TORT (INCLUDING NEGLIGENCE OR OTHERWISE) ARISING IN ANY WAY OUT OF THE USE OF THIS SOFTWARE, EVEN IF ADVISED OF THE POSSIBILITY OF SUCH DAMAGE.

FastPeakFind.m

```
%%% Adi Natan (natan@stanford.edu) // Ver 1.7 // Date: Oct 10th 2013

function  [cent, varargout]=FastPeakFind(d, thres, filt ,edg, res, fid)

% Analyze noisy 2D images and find peaks using local maxima (1 pixel
% resolution) or weighted centroids (sub-pixel resolution).
% The code is designed to be as fast as possible, so I kept it pretty basic.
% The code assumes that the peaks are relatively sparse, test whether there
% is too much pile up and set threshold or user defined filter accordingly.
%
% How the code works:
% In theory, each peak is a smooth point spread function (SPF), like a
% Gaussian of some size, etc. In reality, there is always noise, such as
%"salt and pepper" noise, which typically has a 1 pixel variation.
% Because the peak's PSF is assumed to be larger than 1 pixel, the "true"
% local maximum of that PSF can be obtained if we can get rid of these
% single pixel noise variations. There comes medfilt2, which is a 2D median
% filter that gets rid of "salt and pepper" noise. Next we "smooth" the
% image using conv2, so that with high probability there will be only one
% pixel in each peak that will correspond to the "true" PSF local maximum.
% The weighted centroid approach uses the same image processing, with the
% difference that it just calculated the weighted centroid of each
% connected object that was obtained following the image processing.  While
% this gives sub-pixel resolution, it can miss peaks that are very close to
% each other, and runs slightly slower. Read more about how to treat these
% cases in the relevant code commentes.
%
% Inputs:
% d     The 2D data raw image - assumes a Double\Single-precision
%       floating-point, uint8 or unit16 array. Please note that the code
%       casts the raw image to uint16 if needed.  If the image dynamic range
%       is between 0 and 1, I multiplied to fit uint16. This might not be
%       optimal for generic use, so modify according to your needs.
% thres A number between 0 and max(raw_image(:)) to remove  background
% filt  A filter matrix used to smooth the image. The filter size
%       should correspond the characteristic size of the peaks
% edg   A number>1 for skipping the first few and the last few 'edge' pixels
% res   A handle that switches between two peak finding methods:
%       1 - the local maxima method (default).
%       2 - the weighted centroid sub-pixel resolution method.
%       Note that the latter method takes ~20% more time on average.
% fid   In case the user would like to save the peak positions to a file,
%       the code assumes a "fid = fopen([filename], 'w+');" line in the
%       script that uses this function.
%
%Optional Outputs:
% cent        a 1xN vector of coordinates of peaks (x1,y1,x2,y2,...
% [cent cm]   in addition to cent, cm is a binary matrix  of size(d)
%             with 1's for peak positions. (not supported in the
%             the weighted centroid sub-pixel resolution method)
%
%Example:
%
%   p=FastPeakFind(image);
%   imagesc(image); hold on
%   plot(p(1:2:end),p(2:2:end),'r+')
%
%   Adi Natan (natan@stanford.edu)
%   Ver 1.7 , Date: Oct 10th 2013
%
%% defaults

if (nargin < 1)
    d=uint16(conv2(reshape(single( 2^14*(rand(1,1024*1024)>0.99995) ),[1024 1024])
,fspecial('gaussian', 15,3),'same')+2^8*rand(1024));
    imagesc(d);
end

if ndims(d)>2 %I added this in case one uses imread (JPG\PNG\...).
    d=uint16(rgb2gray(d));
end

if isfloat(d) %For the case the input image is double, casting to uint16 keeps enough
dynamic range while speeds up the code.
    if max(d(:))<=1
```

```
        d =  uint16( d.*2^16./(max(d(:))));
    else
        d = uint16(d);
    end
end

if (nargin < 2)
    thres = (max([min(max(d,[],1))  min(max(d,[],2))])) ;
end

if (nargin < 3)
    filt = (fspecial('gaussian', 7,1)); %if needed modify the filter according to the
expected peaks sizes
end

if (nargin < 4)
    edg =3;
end

if (nargin < 5)
    res = 1;
end

if (nargin < 6)
    savefileflag = false;
else
    savefileflag = true;
end

%% Analyze image
if any(d(:))  ; %for the case of non zero raw image

    d = medfilt2(d,[3,3]);

    % apply threshold
    if isa(d,'uint8')
        d=d.*uint8(d>thres);
    else
        d=d.*uint16(d>thres);
    end

    if any(d(:))   ; %for the case of the image is still non zero

        % smooth image
        d=conv2(single(d),filt,'same') ;

        % Apply again threshold (and change if needed according to SNR)
        d=d.*(d>0.9*thres);

        switch res % switch between local maxima and sub-pixel methods

            case 1 % peak find - using the local maxima approach - 1 pixel resolution

                % d will be noisy on the edges, and also local maxima looks
                % for nearest neighbors so edge must be at least 1. We'll skip 'edge'
                  pixels.
                sd=size(d);
                [x y]=find(d(edg:sd(1)-edg,edg:sd(2)-edg));

                % initialize outputs
                cent=[];%
                cent_map=zeros(sd);

                x=x+edg-1;
                y=y+edg-1;
                for j=1:length(y)
                    if (d(x(j),y(j))>=d(x(j)-1,y(j)-1 )) &&...
                            (d(x(j),y(j))>d(x(j)-1,y(j))) &&...
                            (d(x(j),y(j))>=d(x(j)-1,y(j)+1)) &&...
                            (d(x(j),y(j))>d(x(j),y(j)-1)) && ...
                            (d(x(j),y(j))>d(x(j),y(j)+1)) && ...
                            (d(x(j),y(j))>=d(x(j)+1,y(j)-1)) && ...
                            (d(x(j),y(j))>d(x(j)+1,y(j))) && ...
                            (d(x(j),y(j))>=d(x(j)+1,y(j)+1));

                        %All these alternatives were slower...
                        %if all(reshape( d(x(j),y(j))>=d(x(j)-1:x(j)+1,y(j)-
```

```
                    1:y(j)+1),9,1))
                   %if  d(x(j),y(j)) == max(max(d((x(j)-1):(x(j)+1),(y(j)-
                    1):(y(j)+1))))
                   %if  d(x(j),y(j))  == max(reshape(d(x(j),y(j))  >=  d(x(j)-
                    1:x(j)+1,y(j)-1:y(j)+1),9,1))

                   cent = [cent ;  y(j) ; x(j)];
                   cent_map(x(j),y(j))=cent_map(x(j),y(j))+1; % if a binary matrix
                    output is desired

               end
           end

       case 2 % find weighted centroids of processed image,  sub-pixel resolution.
              % no edg requirement needed.

           % get peaks areas and centroids
           stats = regionprops(logical(d),d,'Area','WeightedCentroid');

           % find reliable peaks by considering only peaks with an area
           % below some limit. The weighted centroid method can be not
           % accurate if peaks are very close to one another, i.e., a
           % single peak will be detected, instead of the real number
           % of peaks. This will result in a much larger area for that
           % peak. At the moment, the code ignores that peak. If that
           % happens often consider a different threshold, or return to
           % the more robust "local maxima" method.
           % To set a proper limit, inspect your data with:
           % hist([stats.Area],min([stats.Area]):max([stats.Area]));
           % to see if the limit I used (mean+2 standard deviations)
           % is an appropriate limit for your data.

           rel_peaks_vec=[stats.Area]<=mean([stats.Area])+2*std([stats.Area]);
           cent=[stats(rel_peaks_vec).WeightedCentroid]';
           cent_map=[];

   end

   if savefileflag
       % previous version used dlmwrite, which can be slower than  fprinf
       %             dlmwrite([filename '.txt'],[cent],   '-append', ...
       %                 'roffset', 0,   'delimiter', '\t', 'newline', 'unix');+

       fprintf(fid, '%f ', cent(:));
       fprintf(fid, '\n');

   end


    else % in case image after threshold is all zeros
        cent=[];
        cent_map=zeros(size(d));
        if nargout>1 ;  varargout{1}=cent_map; end
        return
    end

else % in case raw image is all zeros (dead event)
    cent=[];
    cent_map=zeros(size(d));
    if nargout>1 ;  varargout{1}=cent_map; end
    return
end

%demo mode - no input to the function
if (nargin < 1); colormap(bone);hold on; plot(cent(1:2:end),cent(2:2:end),'rs');hold off;
end

% return binary mask of centroid positions if asked for
if nargout>1 ;  varargout{1}=cent_map; end
```

Licence for remaining Matlab functions

Copyright © 2016, Pierre Mahou
All rights reserved.

Redistribution and use in source and binary forms, with or without modification, are permitted provided that the following conditions are met:

* Redistributions of source code must retain the above copyright notice, this list of conditions and the following disclaimer.
* Redistributions in binary form must reproduce the above copyright notice, this list of conditions and the following disclaimer in the documentation and/or other materials provided with the distribution

THIS SOFTWARE IS PROVIDED BY THE COPYRIGHT HOLDERS AND CONTRIBUTORS "AS IS" AND ANY EXPRESS OR IMPLIED WARRANTIES, INCLUDING, BUT NOT LIMITED TO, THE IMPLIED WARRANTIES OF MERCHANTABILITY AND FITNESS FOR A PARTICULAR PURPOSE
ARE DISCLAIMED. IN NO EVENT SHALL THE COPYRIGHT OWNER OR CONTRIBUTORS BE LIABLE FOR ANY DIRECT, INDIRECT, INCIDENTAL, SPECIAL, EXEMPLARY, OR CONSEQUENTIAL DAMAGES (INCLUDING, BUT NOT LIMITED TO, PROCUREMENT OF SUBSTITUTE GOODS OR SERVICES; LOSS OF USE, DATA, OR PROFITS; OR BUSINESS INTERRUPTION) HOWEVER CAUSED AND ON ANY THEORY OF LIABILITY, WHETHER IN CONTRACT, STRICT LIABILITY, OR TORT (INCLUDING NEGLIGENCE OR OTHERWISE) ARISING IN ANY WAY OUT OF THE USE OF THIS SOFTWARE, EVEN IF ADVISED OF THE
POSSIBILITY OF SUCH DAMAGE.

Fit_Gaussian.m

```
%%% Pierre Mahou // University of Cambridge // 25-05-2016
%%% pierre.mahou@polytechnique.edu
%%% Compare 1D data with a Gaussian function
%%% The fitting parameters are x_param: Amplitude x Offset, Std and
%%% N is the 1D data

function Res_Gauss = Fit_Gaussian(x_Param,Intensity,N)

A = x_Param(1);
B = x_Param(2);
C = x_Param(3);

Res_Gauss  = A*exp(-0.5*(Intensity-B).^2/C^2)-N;
```

Fit_One_Bead_Gauss.m

```
%%% Pierre Mahou // University of Cambridge // 25-05-2016
%%% pierre.mahou@polytechnique.edu
%%% Compare 2D data with a Gaussian function
%%% The fitting parameters are x_param: Amplitude x/y Offset, Std and
%%% Offset. I_Data is the 2D data

function BIC = Fit_One_Bead_Gauss(x_Param,X_Data,I_Data,Param)

S            = Param(1);
Amp          = x_Param(1);
X0           = x_Param(2);
Y0           = x_Param(3);
Sigma        = x_Param(4);
Offset       = x_Param(5);

BIC = Amp*exp(-0.5*((X_Data(:,:,1)-X0).^2+(X_Data(:,:,2)-Y0).^2)/Sigma^2);
BIC = (BIC+Offset-I_Data)/S;

end
```

Fit_One_Ring_Gauss.m

```
%%% Pierre Mahou // University of Cambridge // 25-05-2016
%%% pierre.mahou@polytechnique.edu
%%% Compare 2D data with a Annular function having a gaussian profile
%%% The fitting parameters are x_param: Amplitude x/y Offset, Std and
%%% Offset. I_Data is the 2D data

function BIC = Fit_One_Ring_Gauss(x_Param,X_Data,I_Data,Param)

S            = Param(1);
Width        = Param(2);
Amp          = x_Param(1);
X0           = x_Param(2);
Y0           = x_Param(3);
Radius       = x_Param(4);
Offset       = x_Param(5);

R   = sqrt((X_Data(:,:,1)-X0).^2+(X_Data(:,:,2)-Y0).^2);
BIC = Amp*exp(-0.5*(R-Radius).^2/Width^2);
BIC = (BIC+Offset-I_Data)/S;

end
```

```
%%% Pierre Mahou // University of Cambridge // 25-05-2016
%%% pierre.mahou@polytechnique.edu
%%% Spherical objects in STED data are identified and their positions/widths
%%% are fitted. Objects too dim or too bright can be removed
%%% to exclude artefactual objects in the images. Objects too close to each other can
also be removed
%%% as they tend to bias the results of the fitting.
%%% The fitting function is a 2D gaussian
%%% If Mask_Init = 0, the objects are identified as described above and only the good
%%% candidates are fitted. This strategy can be used to refine gradually
%%% the objects to fit by editing the Guess image i.e. by removing the false positive and
adding
%%% the approximate positions of the "forgotten objects".
%%% Editing can be done for e.g. with Fiji. Each time all the fitt position
%%% at the end of the loop are stored in the image: Rings-Guess-Out-Pos
%%% Results of the fit can be evaluated by inspcting the image: Rings-Out-Fit

clear all
close all
%% Input parameters
N_Img           = 3;                            %%% Image number
Folder_Name     = '03-Vesicles-Mut-48h\';       %%% Foldername
File1           = '-Vesicles-Guess-In';         %%% Filename of the image with the unfused
                                                    vesicles position
File2           = '-STED-Conf-Pix-15nm-Depletion-100-Dwell-20<B5>s'; %%% Filename of the
                                                                         image with the raw
                                                                         data
Extension       = '.tif';
File_Name1      = [Folder_Name num2str(N_Img,'% .2d') File1 Extension]; %%% Mask with the
                                                                          unfused vesicles
                                                                          position (Guess
                                                                          for fitting)
File_Name2      = [Folder_Name num2str(N_Img,'% .2d') File2 Extension]; %%% Raw data with
                                                                          the vesicles
Pix_Size        = 15;           %%% Pixel size in nm
Bead_Size       = 50;           %%% Approximate PSF extension in nm FWHM
Sigma           = Bead_Size/(2*sqrt(2*log(2)))/Pix_Size; %%% standard deviation

Min_Th          = 0;            %%% Threshold for the intensity of the local maxima
                                    (minimum)
Max_Th          = 70;           %%% Threshold for the intensity of the local maxima
                                    (maximum)
N_Hist          = 41;           %%% Bin number for the final histogram
Distance_Bead   = 2;            %%% Minimal distance between two vesicles // Prop. to
                                    Bead_Size
Wbead           = 30;           %%% Window size used for fitting
Mask_Init       = 1;            %%% Use Previous positions as Guess
Threshold       = 8;            %%% Threshold for the local maxima finder
Fit_Th          = 0.5;          %%% Threshold to keep the good fit

%% The STED image is loaded

Img_STED                        = double(imread(File_Name2,'tif',1));
[Sizey,Sizex]                   = size(Img_STED);
[X0_Data(:,:,1),X0_Data(:,:,2)] = meshgrid([1:Sizex],[1:Sizey]);
M                               = median(Img_STED(:));
S                               = std(Img_STED(:));

%% Rough estimation of the vesicle positions. Local maxima comparable or bigger than the
              PSF give good scores
%%% Convolution Mask// Gaussian with the standard deviation of the STED PSF

Kernel        = fspecial('gaussian', 5*round(Sigma),round(Sigma));

%%% If Mask_Init = 1 the vesicles positions are loaded from Img_STED_Position
%%% This strategy can be used to edit manually the false positive/ badly
%%% fitted vesicles by editing Img_STED_Position. Otherwise the approximate vesicles

%%% position are estimated from the raw data (Img_STED)
if Mask_Init ==1
    Img_STED_Position           = double(imread(File_Name1,'tif',1));
    pos                         = FastPeakFind(Img_STED_Position,Threshold,Kernel);
else
    pos                         = FastPeakFind(Img_STED,Threshold,Kernel);
end

Bead_Param    = zeros(size(pos,1)/2,3);
```

```
Bead_Param(:,1) = Img_STED(sub2ind(size(Img_STED),pos(2:2:end),pos(1:2:end)));
Bead_Param(:,2) = pos(1:2:end);
Bead_Param(:,3) = pos(2:2:end);

%%% Display: raw data // raw data with the maxima positions // Distribution of
%%% the maxima intensity
figure(1)
imagesc(Img_STED)
xlabel('Pixels X axis')
ylabel('Pixels Y axis')
title('Raw Data')
set(gca, 'CLim', [0, 20]);
colorbar

figure(2)
imagesc(Img_STED); hold on
plot(pos(1:2:end),pos(2:2:end),'r+')
hold off
xlabel('Pixels X axis')
ylabel('Pixels Y axis')
title('Raw Data with the positions of the local maxima')
set(gca, 'CLim', [0, 20]);
colorbar

figure(3)
hist(squeeze(Bead_Param(:,1)),N_Hist)
xlabel('Intensity (a.u.)')
ylabel('Counts')
title('Distribution of the local maxima intensity')

%% Vesicles too dim and too bright are excluded
Amp             = squeeze(Bead_Param(:,1));
Posx            = squeeze(Bead_Param(:,2));
Posy            = squeeze(Bead_Param(:,3));

Ind             = find((Amp>Min_Th).*(Amp<Max_Th));
Amp             = Amp(Ind);
Posx            = Posx(Ind);
Posy            = Posy(Ind);

[Sort_Amp,IX]   = sort(Amp);
Sort_Posx       = Posx(IX);
Sort_Posy       = Posy(IX);
%% Vesicles too close to each other are excluded
count           = 0;
One_Beads       = 0;
One_Beads_Param = [];
N_One_Beads     = floor(size(Amp,1));
One_Beads_Fit   = zeros(Sizey,Sizex);
Width           = 0;
R2              = 0;

for p =1:N_One_Beads-1
Dist_Beads     = sqrt((Sort_Posx-Sort_Posx(p)).^2+(Sort_Posy-Sort_Posy(p)).^2);
Dist_Beads     = sort(Dist_Beads);
    if min(Dist_Beads(2:end))> (Distance_Bead*Bead_Size/Pix_Size)
        One_Beads_Param = [One_Beads_Param;Sort_Amp(p),Sort_Posx(p),Sort_Posy(p)];
        count = count+1;
    else
    end
end

One_Beads_Param = [One_Beads_Param;Sort_Amp(end),Sort_Posx(end),Sort_Posy(end)];

%%% Display: raw data with the positions of the selected vesicles
figure(4)
imagesc(Img_STED); hold on
plot(One_Beads_Param(:,2),One_Beads_Param(:,3),'r+')
hold off
xlabel('Pixels X axis')
ylabel('Pixels Y axis')
title('Raw Data with the selected local maxima')

%%% Sizes and position of the unfused vesicles are fitted one by one
%%% Only the selected vesicles are fitted

[X(:,:,1),X(:,:,2)] = meshgrid((1:Wbead),(1:Wbead));
```

```
for k=1:count+1
    OneBeadArea    = floor([min(max(1,One_Beads_Param(k,3)-(Wbead-1)/2),Sizey-
          Wbead+1),min(max(1,One_Beads_Param(k,3)-(Wbead-1)/2)+Wbead-1,Sizey),...
        min(max(1,One_Beads_Param(k,2)-(Wbead-1)/2),Sizex-
          Wbead+1),min(max(1,One_Beads_Param(k,2)-(Wbead-1)/2)+Wbead-1,Sizex)]);
    BeadK          =
          Img_STED(OneBeadArea(1):OneBeadArea(2),OneBeadArea(3):OneBeadArea(4));

    Init_Param     = [One_Beads_Param(k,1),One_Beads_Param(k,2)-
          OneBeadArea(3)+1,One_Beads_Param(k,3)-OneBeadArea(1)+1,Sigma,M];
    [fp,resnorm]   = lsqnonlin(@Fit_One_Bead_Gauss,Init_Param,[0 0 0 0
          0],[max(2*One_Beads_Param(k,1),1) Wbead Wbead 4*Sigma 3*M],[],X,BeadK,[S M]);
    Width(k)       = Pix_Size*(2*sqrt(2*log(2)))*fp(end-1);
    R2(k)          = 1-S^2*resnorm/sum(sum((BeadK-M).^2));

    if R2(k)>Fit_Th
    One_Beads_Fit = One_Beads_Fit+fp(1)*exp(-0.5*((X0_Data(:,:,1)-(fp(2)+OneBeadArea(3)-
          1)).^2+(X0_Data(:,:,2)-(fp(3)+OneBeadArea(1)-1)).^2)/fp(4)^2);
    Img_STED       = Img_STED-round(fp(1)*exp(-0.5*((X0_Data(:,:,1)-(fp(2)+OneBeadArea(3)-
          1)).^2+(X0_Data(:,:,2)-(fp(3)+OneBeadArea(1)-1)).^2)/fp(4)^2));
    Ind_Neg        = find(Img_STED<0);
    Img_STED(Ind_Neg) = M+S*randn(length(Ind_Neg),1);

    hFig = figure(5);
    set(hFig, 'Position', [100, 050, 1049, 250])
    subplot(1,3,1)
    imagesc(BeadK)
    hold on; plot(Init_Param(2),Init_Param(3),'+k'); hold off;
    colorbar; xlabel('Pixels X axis'); ylabel('Pixels Y axis');

    subplot(1,3,2)
    imagesc(fp(1)*exp(-0.5*((X(:,:,1)-fp(2)).^2+(X(:,:,2)-fp(3)).^2)/fp(4)^2))
    hold on; plot(Init_Param(2),Init_Param(3),'+k'); hold off
    colorbar; xlabel('Pixels X axis'); ylabel('Pixels Y axis');

    subplot(1,3,3)
    imagesc(BeadK-fp(1)*exp(-0.5*((X(:,:,1)-fp(2)).^2+(X(:,:,2)-fp(3)).^2)/fp(4)^2))
    hold on; plot(Init_Param(2),Init_Param(3),'+k'); hold off
    colorbar; xlabel('Pixels X axis'); ylabel('Pixels Y axis');
    drawnow update
    end

end

%% Only the good fit are kept
Ind_Good     = find(R2>Fit_Th);
Width_Good   = Width(Ind_Good);

%% Size distribution of the unfused vesicles
[N_Res Res]         = hist(Width_Good,(0:5:300));
initpar             = [max(N_Res) mean(Width_Good) std(Width_Good)];
[outpar,resnorm]    = lsqnonlin(@Fit_Gaussian,initpar,[],[],[],Res,N_Res);
Resint              = 0:0.01:Res(end);
N_Resint            = outpar(1)*exp(-0.5*(Resint-outpar(2)).^2/outpar(3)^2);

figure(6)
hold on
bar(Res,N_Res)
plot(Resint,N_Resint,'r','LineWidth',3)
hold off; axis([0 300 0 180])
xlabel('Estimated diameter (nm)'); ylabel('Counts')
hold off; box on; grid on

%% Sizes and positions of the unfused vesicles are saved
imwrite(uint16(One_Beads_Fit),[Folder_Name num2str(N_Img,'% .2d') '-Vesicles-Guess-Out'
     Extension],'tiff');
imwrite(uint16(Img_STED),[Folder_Name num2str(N_Img,'% .2d') '-Unfitted-Vesicles'
     Extension],'tiff');

f = fopen([Folder_Name num2str(N_Img,'% .2d') '-STED-Diameter-Vesicles.txt'], 'w');
fprintf(f, '%s\t%s\r\n', 'N Vesicles', 'Diameter nm');
for k = 1:length(Width)
    fprintf(f, '%d\t%d\r\n', k, Width(k));
end
fclose(f);
```

```
figure(7)
plot(R2,'o')
```

Ring_Finder_Affine.m

```
%%% Pierre Mahou // University of Cambridge // 25-05-2016 //
%%% pierre.mahou@polytechnique.edu
%%% Annular objects in STED data are identified and their positions/widths
%%% are fitted.
%%% The fitting function is a annulus with a Gaussian radial profile
%%% If Mask_Init = 1, the script loads an image where a Guess of the objects positions
are stored (Img_Ring_Pos).
%%% In this case only the objects stored in the Guess are fitted
%%% If Mask_Init = 0, the objects are identified as described above and only the good
%%% candidates are fitted. This strategy can be used to refine gradually
%%% the objects to fit by editing the Guess image i.e. by removing the false positive and
adding
%%% the approximate positions of the "forgotten objects".
%%% Editing can be done for e.g. with Fiji. Each time all the fitt position
%%% at the end of the loop are stored in the image: Rings-Guess-Out-Pos
%%% Results of the fit can be evaluated by inspcting the image: Rings-Out-Fit

clear all
close all
%% Input parameters
N_Img           = 3;                          %%% Image number
Folder_Name     = '03-Vesicles-Mut-48h\';     %%% Foldername
File1           = '-Rings-Guess-In-Pos';      %%% Filename of the image with the fused
                                                   vesicles position
File2           = '-Unfitted-Vesicles';       %%% Filename of the image with the fused
                                                   vesicles
Extension       = '.tif';
File_Name1      = [Folder_Name num2str(N_Img,'% .2d') File1 Extension]; %%% Mask with the
                                                                            fused
                                                                            positions
                                                                            (Guess for
                                                                            fitting)
File_Name2      = [Folder_Name num2str(N_Img,'% .2d') File2 Extension]; %%% Raw data with
                                                                            the Rings
Pix_Size        = 15;           %%% Pixel size in nm
Bead_Size       = 50;           %%% Approximate PSF extension in nm FWHM
Sigma           = Bead_Size/(2*sqrt(2*log(2)))/Pix_Size; %%% Standard deviation of the
                                                             PSF
Width           = 50/(2*sqrt(2*log(2)))/Pix_Size; %%% Width of the ring used for fitting
Threshold       = 150;                 %%% Threshold for the local maxima finder
Wring           = 50;                  %%% Window size used for fitting
Radius          = 7;                   %%% Radius, initial value for fitting
N_Ker           = 31;                  %%% Size of the Kernel

Mask_Init       = 1;                   %%% Use Previous positions as Guess
Fit_Th          = 0.15;                %%% Threshold to keep the good fit

%% Load the STED image with the large vesicles only

Img_STED_Ring                   = double(imread(File_Name2,'tif',1));
[Sizey,Sizex]                   = size(Img_STED_Ring);
[X0_Data(:,:,1),X0_Data(:,:,2)] = meshgrid([1:Sizex],[1:Sizey]);
M                               = median(Img_STED_Ring(:));
S                               = std(Img_STED_Ring(:));

%% Find the approximate positions // Guess for fitting
%%% Local maxima after convolution with an annulus function are kept
%%% Annulus objects give good scores
%%% Convolution Mask// Annulus function with the width of the STED PSF and
%%% a user defined radius
if Mask_Init ==1
    Img_Ring_Pos  = double(imread(File_Name1,'tif',1));
    Kernel        = fspecial('gaussian', 5*round(Width),round(Width));
    pos_Ring      = FastPeakFind(Img_Ring_Pos,0,Kernel);
else
    [X_Ker, Y_Ker]= meshgrid((1:N_Ker),(1:N_Ker));
    R_Ker         = sqrt((X_Ker-(N_Ker+1)/2).^2+(Y_Ker-(N_Ker+1)/2).^2);
    Kernel        = (R_Ker<Radius+2)-(R_Ker<Radius);
    Kernel        = (R_Ker<Radius+2)-(R_Ker<Radius);
    Corr_Ring     = conv2(Img_STED_Ring, Kernel,'same');
    Kernel        = fspecial('gaussian', 5*Radius,Radius);
    pos_Ring      = FastPeakFind(Corr_Ring,Threshold,Kernel);
end

%%% Display: raw data with rings only// raw data with the maxima positions
```

```
figure(1)
imagesc(Img_STED_Ring)
xlabel('Pixels X axis')
ylabel('Pixels Y axis')
title('Raw Data with rings only')
set(gca, 'CLim', [0, 20]);
colorbar

figure(2)
imagesc(Img_STED_Ring); hold on
plot(pos_Ring(1:2:end),pos_Ring(2:2:end),'r+')
hold off;
xlabel('Pixels X axis');ylabel('Pixels Y axis')
title('Raw Data with the positions of the local maxima for the rings')
set(gca, 'CLim', [0, 20]);
colorbar

%% Sizes and position of the fused vesicles are fitted one by one

Ring_Param          = zeros(size(pos_Ring,1)/2,3);
Ring_Param(:,1)     = 0;
Ring_Param(:,2)     = pos_Ring(1:2:end);
Ring_Param(:,3)     = pos_Ring(2:2:end);
[X(:,:,1),X(:,:,2)] = meshgrid((1:Wring),(1:Wring));

Ring_Fit        = 0;
Img_Pos         = 0;
Img_Ring_Fit    = 0;
Diam            = [];
R2              = 0;

for k=1:size(Ring_Param,1)
    RingArea        = floor([min(max(1,Ring_Param(k,3)-(Wring-1)/2),Sizey-
         Wring+1),min(max(1,Ring_Param(k,3)-(Wring-1)/2)+Wring-1,Sizey),...
        min(max(1,Ring_Param(k,2)-(Wring-1)/2),Sizex-Wring+1),min(max(1,Ring_Param(k,2)-
          (Wring-1)/2)+Wring-1,Sizex)]);
    RingK           = Img_STED_Ring(RingArea(1):RingArea(2),RingArea(3):RingArea(4));
    Ring_Param(k,1) = max(RingK(:));
    Init_Param      = [Ring_Param(k,1),Ring_Param(k,2)-RingArea(3)+1,Ring_Param(k,3)-
         RingArea(1)+1,Radius,M];
    [fp,resnorm]    = lsqnonlin(@Fit_One_Ring_Gauss,Init_Param,[0 0 0 0
         0],[max(2*Ring_Param(k,1),1) Wring Wring 4*Radius 3*M],[],X,RingK,[S Width]);
    R               = sqrt((X(:,:,1)-fp(2)).^2+(X(:,:,2)-fp(3)).^2);
    Ring_Fit        = Ring_Fit+fp(1)*exp(-0.5*(R-fp(4)).^2/Width^2);
    Diam            = [Diam Pix_Size*2*fp(4)];
    R2(k)         = 1-S^2*resnorm/sum(sum((RingK-M).^2));

    if R2(k)>Fit_Th
    R0              = sqrt((X0_Data(:,:,1)-(fp(2)+RingArea(3)-1)).^2+(X0_Data(:,:,2)-
          (fp(3)+RingArea(1)-1)).^2);
    Img_STED_Ring   = Img_STED_Ring-round(fp(1)*exp(-0.5*(R0-fp(4)).^2/Width^2));
    Ind_Neg         = find(Img_STED_Ring<0);
    Img_STED_Ring(Ind_Neg) = M+S*randn(length(Ind_Neg),1);
    Img_Pos         = Img_Pos+(2^8-1)*exp(-0.5*((X0_Data(:,:,1)-(fp(2)+RingArea(3)-
         1)).^2+(X0_Data(:,:,2)-(fp(3)+RingArea(1)-1)).^2)/Width^2);
    Img_Ring_Fit    = Img_Ring_Fit+fp(1)*exp(-0.5*(R0-fp(4)).^2/Width^2);

    hFig = figure(3);
    set(hFig, 'Position', [100, 050, 1049, 250])
    subplot(1,3,1)
    imagesc(RingK)
    hold on; plot(Init_Param(2),Init_Param(3),'+k'); hold off;
    colorbar; xlabel('Pixels X axis'); ylabel('Pixels Y axis');

    subplot(1,3,2)
    imagesc(fp(1)*exp(-0.5*(R-fp(4)).^2/Width^2))
    hold on; plot(Init_Param(2),Init_Param(3),'+k'); hold off
    colorbar; xlabel('Pixels X axis'); ylabel('Pixels Y axis');

    subplot(1,3,3)
    imagesc(RingK-round(fp(1)*exp(-0.5*(R-fp(4)).^2/Width^2)))
    hold on; plot(Init_Param(2),Init_Param(3),'+k'); hold off
    colorbar; xlabel('Pixels X axis'); ylabel('Pixels Y axis');
    drawnow update
    end
end
```

```
%% Only the good fits are kept
Ind_Good     = find(R2>Fit_Th);
Diam_Good    = Diam(Ind_Good);
[N_Diam Res] = hist(Diam_Good,(0:5:300));

%% Size distribution of the fused vesicles
figure(4)
hold on
bar(Res,N_Diam)
hold off
xlabel('Estimated diameter (nm)'); ylabel('Counts')
hold off; box on; grid on; axis([0 300 0 80])

%% Sizes and positions of the fused vesicles are saved
imwrite(uint16(Img_Pos),[Folder_Name num2str(N_Img,'% .2d') '-Rings-Guess-Out-Pos'
Extension],'tiff');
imwrite(uint16(Img_Ring_Fit),[Folder_Name num2str(N_Img,'% .2d') '-Rings-Out-Fit'
Extension],'tiff');

f = fopen([Folder_Name num2str(N_Img,'% .2d') '-STED-Diameter-Ring.txt'], 'w');
fprintf(f, '%s\t%s\r\n', 'N Vesicles', 'Diameter nm');
for k = 1:length(Diam)
    fprintf(f, '%d\t%d\r\n', k, Diam(k));
end
fclose(f);

figure(7)
plot(R2,'o')
```
