# Supplementary material for: Structural basis of synaptic vesicle assembly promoted by α-synuclein
Source: Nat Commun. 2016 Sep 19;7:12563. doi: 10.1038/ncomms12563 (PMC5031799; doi:10.1038/ncomms12563)
Supplement: Supplementary Data 2 — Instructions for obtaining rapidSTORMM [file ncomms12563-s3.pdf]

Supplementary Data 2. rapidSTORMM.

rapidSTORMM is an open access software.

It is available at https://idefix.biozentrum.uni-wuerzburg.de/software/rapidSTORM/
